# Tritium Accommodation and Diffusion in Li_8_PbO_6_ from First-Principles Simulations

**DOI:** 10.1021/acs.jpcc.4c08016

**Published:** 2025-01-14

**Authors:** Andrew W. Davies, Samuel T. Murphy

**Affiliations:** Department of Engineering, Lancaster University, Bailrigg, Lancaster LA1 4YW, U.K.

## Abstract

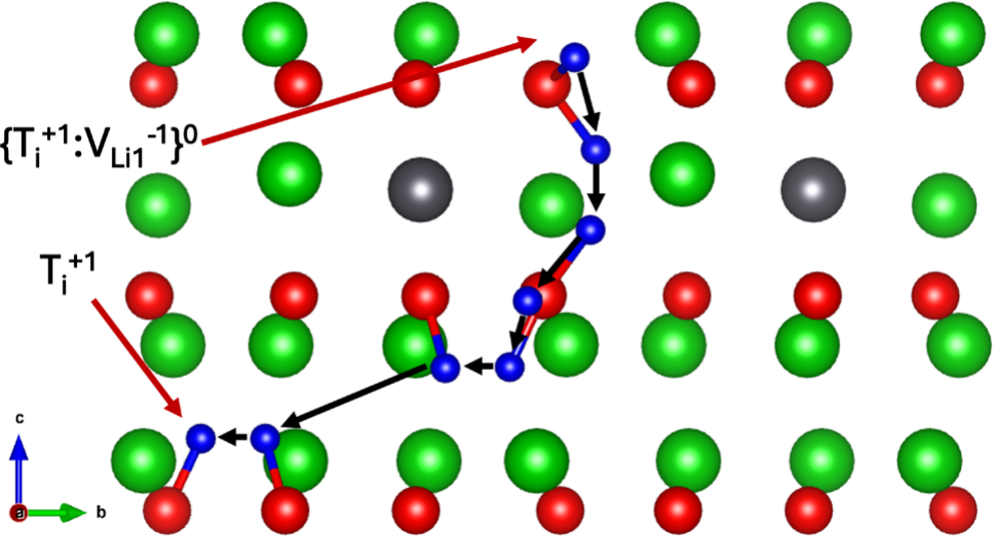

Li_8_PbO_6_ has been proposed as an alternative candidate breeding blanket material for use in fusion reactors. As lithium is burned inside the blanket, tritium is produced within the ceramic matrix until it reaches the surface, from where it is recovered by isotope exchange reactions. To fully understand the tritium recovery process, it is essential to understand how tritium is accommodated in the fuel and subsequently migrates to the surface. Therefore, in this work, we employ density functional theory (DFT) to examine tritium accommodation in Li_8_PbO_6_. We then used the nudged elastic band (NEB) method to understand the mechanisms for the migration of tritium-accommodating defects in Li_8_PbO_6_. We have found tritium is more likely bind to an oxygen ion and form a hydroxyl than exist in the traditional interstitial sites. We predict the barriers for migration of tritium interstitials to be anisotropic, with barriers of 0.27 and 0.69 eV along the *xy*-plane and through the *z*-axis, respectively. The barrier for escape from a lithium vacancy trapping site we found to be in the range of 0.76–0.85 eV, and an activation energy range of 0.67–1.18 eV for the migration of the trapping site as a whole. Due to the low migration energies found, we predict that aging of the blanket will have a lower significance on tritium release compared to other leading candidate materials.

## Introduction

With the growing demand for low-carbon energy sources, nuclear fusion presents an attractive alternative due to the combined reduction of nuclear waste and safety issues compared to modern-day fission reactors. One of the major barriers to maintaining a fleet of reactors is the lack of naturally occurring tritium, which is required for the D-T reaction (eq 1), i.e.,
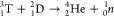
1

This paucity of naturally occurring tritium is due to a combination of being generated by a rare event involving cosmic rays and an intrinsically low half-life of 12.32 years.^1,2^ Consequently, it is imperative to generate the necessary tritium artificially. This will be done *in situ* in the reactor itself, using a “tritium breeding blanket,” which surrounds the walls of the reactor chamber. The blanket will generate tritium by utilizing the transmutation of lithium, driven by the high energy neutrons ejected from the D-T fusion plasma as illustrated in eqs 2 and 3:
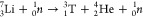
2
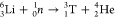
3

Theoretically, for the blanket to fulfill the tritium demand required to maintain a fleet of fusion reactors, the number of tritium ions extracted from the blanket per fusion reaction occurring in the plasma must be greater than 1. In practice, the tritium breeding ratio (TBR) should be >1.1 to account for neutron losses, tritium loss due to radioactive decay and seepage into other reactor components.^3^ To reach such a high TBR, it is necessary to incorporate a neutron multiplier, typically beryllium, to increase the number of neutrons available to transmute lithium. Unfortunately, the introduction of beryllium presents its own issues; beryllium is not only a relatively rare material but also contains trace impurities of uranium, which are difficult to remove and in the presence of a high neutron flux (such as that generated by the plasma), produces long-lived radioactive byproducts.^4^

Solid breeder materials being developed for use in the ITER and DEMO projects are the ceramics, lithium metatitanate (Li_2_TiO_3_),^5^ and lithium orthosilicate (Li_4_SiO_4_).^6^ These materials have their own limitations, and both require a beryllium multiplier to achieve maximum potential TBRs of the order of 1.1–1.15, barely sufficient to maintain a fleet of reactors. Recently, Hernández and Pereslavtsev explored the possibility of utilizing octolithium compounds, such as octalithium plumbate (Li_8_PbO_6_) due to their high lithium densities, which could potentially offer high TBRs^7^ and reduce dependence on a beryllium multiplier.^8^ For Li_8_PbO_6_, the incorporation of the lead multiplier in the materials structure makes it the leading octolithium compound, from a neutronics perspective.

In addition to its attractiveness from a neutronics perspective, Hayashi et al.^9^ suggest Li_8_PbO_6_ has favorable tritium release characteristics. Once formed and accommodated in the crystal, the tritium must diffuse through the bulk material until it reaches a grain boundary where it can then be transported to the surface and extracted by a helium purge gas. The rate-limiting step for tritium recovery is the migration of tritium through the bulk until it reaches a grain boundary. Hayashi et al.^9^ calculated an activation energy of 0.78 eV for tritium release from Li_8_PbO_6_, compared to 0.4–0.88 eV^10−12^ for Li_2_TiO_3_ and 0.63–1.5 eV for Li_4_SiO_4_.^13,14^ While Hayashi et al.^9^ were unable to identify the exact mechanisms for tritium release, they were able to demonstrate that tritium primarily takes the form of T^+1^.

Atomistic simulation studies of migration pathways of tritium in other ceramic breeding materials, such as Li_2_TiO_3_, have utilized a point defect model in order to predict defect migration barriers.^15^ As observed in other studies, the stoichiometry of the host ceramic likely determines the mode of tritium transport. If there is a lithium excess, tritium is expected to migrate almost exclusively as an interstitial hopping between oxygen ions as a hydroxyl species. If there is a depletion of lithium (which is expected to occur as the material ages), tritium is more likely to form a {}^×^ defect complex, which suggests a change in the tritium release performance during operation.

In this work, density functional theory (DFT) is used to examine the accommodation of tritium according to processing and operational conditions, followed by characterization of tritium migration pathways through the bulk crystal with in the presence of lithium vacancy defects.

## Crystallography and Defect Notation

**Figure 1:**
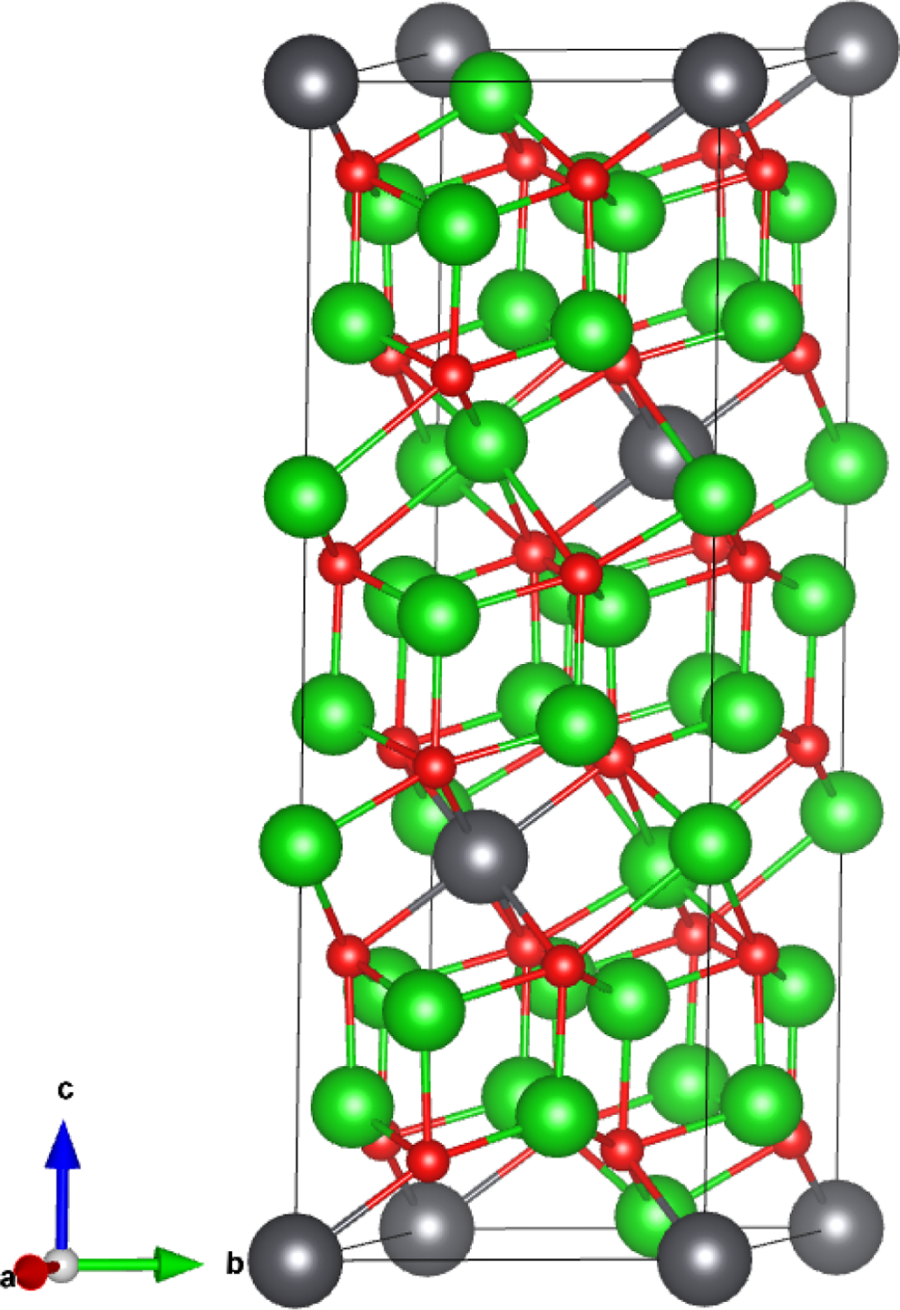
45-atom unitcell of Li_8_PbO_6_. Gray ions indicate lead, red ions indicate oxygen, and green ions indicate lithium.

As illustrated in Figure 1, Li_8_PbO_6_ crystallizes into the R3̅H [148] space group and forms a complex layered structure, alternating between a mixed lithium–lead and pure lithium layers in the sequence PbLi_2_–O_3_–Li_3_–Li_3_-–O_3_. There are two symmetrically distinct lithium sites, a tetrahedrally coordinated site existing exclusively in the pure lithium layer (18*f* Wyckoff site) and an octahedrally coordinated site in the mixed lithium–lead layer (6*c*), which will be distinguished for the rest of this work using the labels Li1 and Li2 for the tetrahedrally and octahedrally coordinated sites, respectively. The oxygen and lead ions occupy the 18*f* and 3*a* sites, respectively.

Point defects will be represented using a modified form of the Kroger-Vink notation,^16^, where *A* represents the atom type, or *V* if a vacancy, *S* is the site, or *i* if an interstitial and *c* is the relative charge. Note that in the original notation • was used for a positive charge and ^′^’s for a negative charge; however, numbers are employed here for ease of reading. These are not, however, the charges on the ions themselves.^17^

## Methodology

### Defect Formalism

Due to the inherent role of the breeding blanket, the ceramic is expected to become more deficient in lithium as it undergoes transmutation throughout the operating lifetime of the material, as well as experiencing intense irradiation by high-energy neutrons. These factors will likely impact the level of tritium retention as the material ages. Therefore, prior to the investigation of migration pathways of tritium, we explore the defect population due to the incorporation of nonstoichiometry.

#### Defect Concentrations

Considering only point and electronic defects, the charge neutrality condition for a crystal can be met according to eq 4:
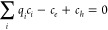
4where, the concentration of a point defect *i* with a charge *q*_*i*_ is given by *c*_*i*_. *c*_*e*_ and *c*_*h*_ represent the concentration of electronic defects in the crystal (i.e., electrons and holes in the conduction and valence bands, respectively), and can be calculated using Fermi–Dirac statistics according to eqs 5 and 6:
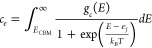
5and
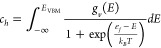
6where, *E*_CBM_ is the energy of the conduction band minimum, g_c_(E) and g_v_(E) are the electronic density of states calculated using the hybrid HSE06 functional of Heyd, Scuseria, and Ernzerhof,^18^ and are taken from our previous work exploring the fundamental properties of Li_8_PbO_6_,^19^ ε_*f*_ is the Fermi energy, and *E*_VBM_ represents the energy at the valence band maximum.

The formation energy for a point defect *i* can be approximated to the change in internal energy required to form the defect using eq 7:
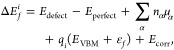
7where *E*_defect_ – *E*_perfect_ is the difference in lattice energies between the perfect and defective crystal calculated using DFT. *n*_α_ is the number of atoms of species α added/removed from the crystal, and μ_α_ is the chemical potential of α. q_*i*_(*E*_VBM_ + ε_*f*_) represents the impact of the charge of the defect where q_*i*_ is the charge state. *E*_corr_ is a finite size correction discussed in further detail later.

As we will be examining the concentration of defects under multiple different processing conditions, the chemical potentials for the constituents of Li_8_PbO_6_ shall be derived from the Li_2_O and PbO_2_ solid binary oxides used to form Li_8_PbO_6_^20^ according to eq 8:
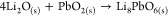
8

The chemical potential of the host Li_8_PbO_6_ must equal the sum of the chemical potentials of the constituent binary oxides as illustrated in eq 9:
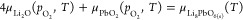
9where  and  represent the chemical potentials of Li_2_O and PbO_2_,  is the chemical potential of solid Li_8_PbO_6_, and  is the oxygen partial pressure.

Typically, at low temperatures, it is safe to assume any vibrational contributions will be negligible, i.e., . However, due to the high operating temperatures breeder blankets are expected to operate at, it may not be appropriate to apply such an assumption. In previous work, we showed that there is a non-negligible impact on the predicted intrinsic defect chemistry of Li_8_PbO_6_ if vibrational contributions to energies of the reference states are included than when standard, energy-minimized, internal energies are used for the chemical potentials of the solid compounds, i.e., , , and . Temperature contributions to energies of the reference states used to substitute the DFT internal energies are taken from previous work^21^ using the quasi-harmonic approximation (QHA) in the Phonopy software,^22^ and for Li_2_O, the Gibbs free energy is taken from works by Chase^23^ and Johnston and Bauer,^24^ due to the presence of phonon–phonon interactions present in FCC lattice structures.^25^ Where appropriate, the defect chemistry is predicted up to 1200 K as phonon–phonon interactions become a prominent feature at very high temperatures, which is unsuitable for QHA.

At equilibrium, the chemical potentials of the constituent solid oxides in the crystal act as an upper bound for the constituent chemical potentials. If the chemical potential exceeds this, i.e., if  exceeds that of  (Li_2_O-rich conditions), an Li_2_O precipitate will begin to form. A lower bound for the chemical potential can be found by assuming that the chemical potential of the opposing binary compound is at their upper bound. Equation 10 shows the Li_2_O-poor case:
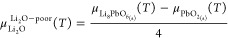
10

The constituent binary oxides can be further broken down to determine the chemical potentials of the Li, Pb, and O subcomponents as illustrated in eq 11:
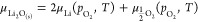
11where  and  are the chemical potentials of Li and O in Li_2_O. Due to the well-known errors of semilocal DFT functionals for determining the energy of the O_2_ dimer, the chemical potential of O_2_ is not derived directly from DFT. Instead, we utilize the method first suggested by Finnis et al.,^26^ which references , the experimental formation energy of Li_2_O. The chemical potential of oxygen at standard pressure and temperature can be obtained from eq 12:
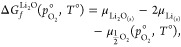
12where  is taken from Chase^27^ as −6.205 eV ( is also taken from Chase as −2.845 eV). As  is calculated twice (once for each binary oxide), a weighted average for  is taken according to the fraction of Li_2_O and PbO_2_ defined in the chemical potential scheme.^28^

The chemical potential of  is extrapolated from  using eq 13:
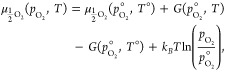
13where  is determined from the experimental heat capacity for the O_2_ molecule, taken from the NIST Chemistry WebBook.^29^

### Computational Procedure

All DFT simulations in this work were performed using the Vienna A*b initio* Simulation Package (VASP—version 5.4.4) plane-wave pseudopotential code,^30^ using projector augmented wave (PAW) pseudopotentials^31^ and utilizing the generalized gradient approximation (GGA) functional of Perdew, Burke, and Ernzerhof (PBE).^32^ Integration over the Brillouin zone was performed on a 45-atom unit cell as shown in Figure 1 using a 6 × 6 × 2 Monkhorst–Pack grid^33^ with a separation between *k*-points of 0.0316 ×2π Å^–1^ along *x* and *y* and 0.0344 ×2π Å ^–1^ along the *z*-axis. A plane-wave cutoff energy of 650 eV was used, and structural convergence is assumed to be complete when the forces on all atoms fell below 0.01 eV Å ^–1^. As defects are introduced into the crystal, a sufficiently sized supercell should be used. In this work, a 2 × 2 × 1 180-atom supercell created from the 45-atom unitcell shown in Crystallography and Defect Notation section is used, with a 3 × 3 × 2 *k*-point grid. Defect charges are modeled by introducing/removing electrons into/from the supercell.

#### Finite Size Effects

The Coulombic interaction between a defect and its periodic images, as well as the interaction with the charge-neutralizing background, results in an artifact in the calculated defect formation energy. As these interactions are slow to converge with supercell size, it is appropriate to apply a finite size correction to compensate for this error.

In this paper, we use the anisotropic screening correction developed by Kumagai and Oba,^34^ building on the correction developed by Freysoldt, Neugebauer, and Van de Walle (FNV)^35^ by using atomic site potentials rather than planar-averaged electrostatic potentials. The finite size correction *E*_corr_ can be calculated using eqs 14–16:
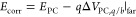
14
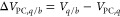
15
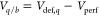
16where the defect-induced potential V_q/b_ is the difference in electronic potentials between a perfect (*V*_perf_) and defective unit cell (*V*_def,q_), Δ*V*_PC,q/b_ is the difference between the defect-induced potential and the point charge potential *V*_PC,q_ and Δ*V*_PC,q/b_|_far_ is simply Δ*V*_PC,q/b_ measured far from the defect.

*E*_*PC*_ is a point charge correction, calculated using eq 17:
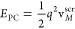
17where  is the screened Madelung potential. As the dielectric tensor of Li_8_PbO_6_ is anisotropic, the tensorial approach to calculating  described by Murphy and Hine is used.^36^ The dielectric tensor for Li_8_PbO_6_ is taken from previous work.^19^

### Nudged Elastic Band

Activation energy barriers for defect migration were calculated using the climbing Nudged Elastic Band method (cNEB).^37,38^ NEB is a method for finding minimum energy paths by following the saddle points between reactants. A series of images of the defect along the migration pathway is generated and optimized, and a tangential spring force is applied to each image to find the optimal pathway between images. A small spring force is applied between images to ensure they are as equally spaced as possible. cNEB adopts a different approach by instead pushing the image with the highest energy upward toward the saddle point. This is achieved by inverting the tangential force at this image such that it tries to maximize its energy along the pathway. As the maximized image does not feel the same spring force between neighboring images, the images are no longer evenly spaced like in regular NEB.

For each migration pathway considered in this work, 6 images were generated between the initial and final sites. The spring constant used was kept as the default −5 eV/Å^2^. A consequence of linear interpolation in procuring the intermediary images between the initial and final states is the likelihood of a defect within an intermediary image being placed into a suboptimal position (e.g., an interstitial defect in close proximity to another ion). Thus, the requirement for structural convergence is modified so that instead of requiring the total forces on all atoms to fall below 0.01 eV Å ^–1^, this is relaxed to 0.08 eV Å ^–1^.

## Results and Discussion

### Tritium Defect Formation Energies

**Figure 2:**
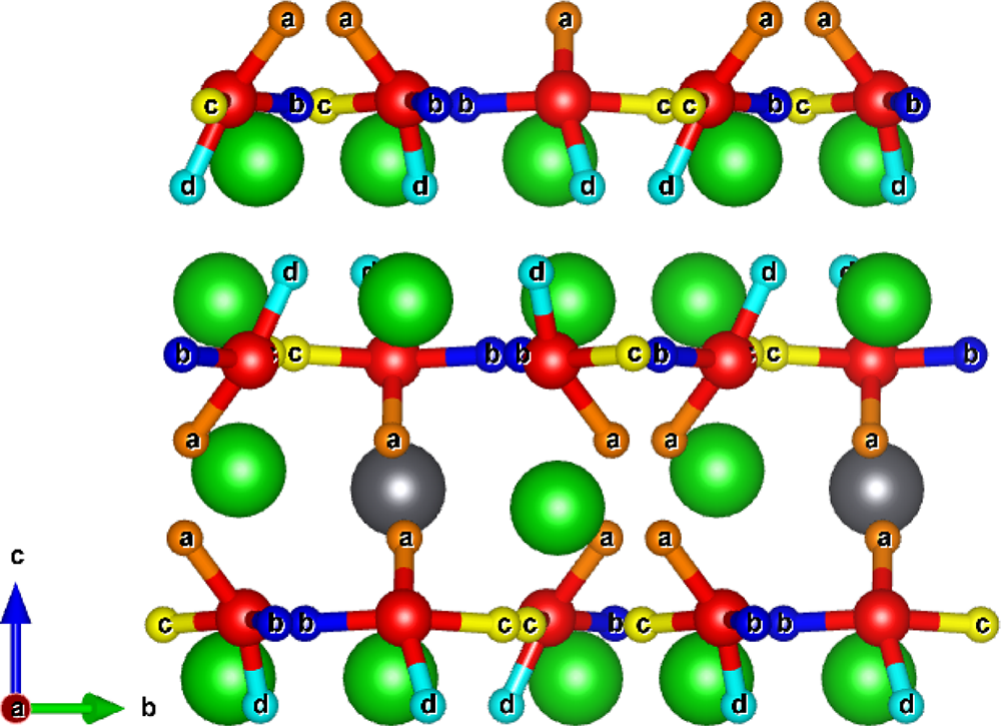
Location of tritium interstitial sites in Li_8_PbO_6_. Orange, blue, yellow, and teal ions represent tritium interstitial sites a - *d* respectively.

**Figure 3:**
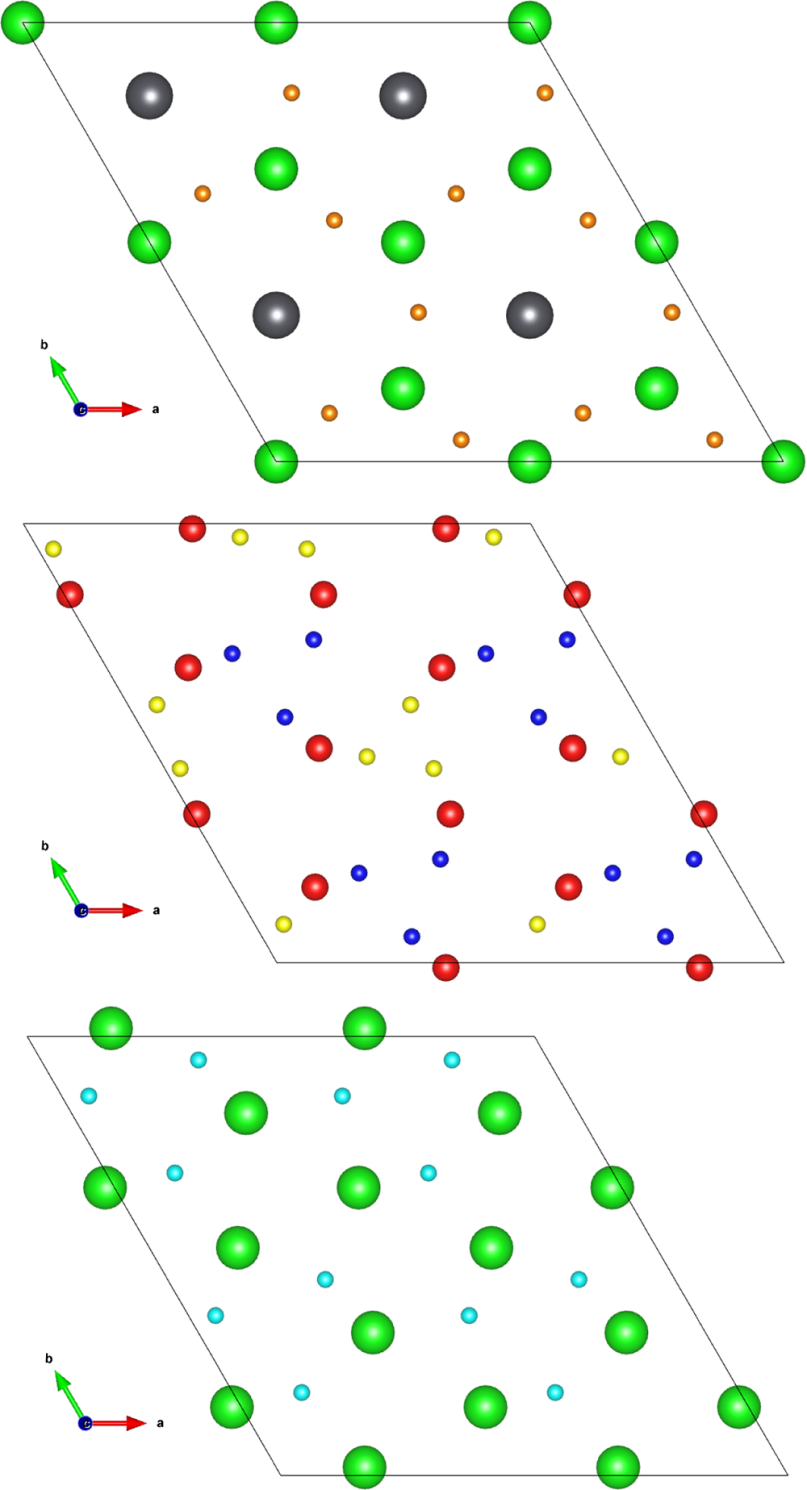
Planar view of possible tritium interstitial sites. Top: Mixed Li–Pb plane and occupation of T at interstitial site *A*, represented by orange-colored ions. Middle: Pure O plane and occupation of T at interstitial sites *b* and *c*, represented by blue and yellow-colored ions, respectively. Bottom: Pure Li plane and occupation of T at interstitial site *d*, represented by teal-colored ions.

Four symmetrically distinct interstitial sites for tritium were found in the vicinity of the Li1 site (labeled *a*—*d* in Figure 2) by performing a geometry optimization of a charged tritium ion at the midpoint of the connecting Li–O bonds and allowing the tritium to form a hydroxyl with a neighboring oxygen ion. Previous work examining the 6 Li–O bonds surrounding the Li2 site showed that irrespective of the starting point, all initial arrangements relaxed to give the *a* configuration.^19^ Site a occupies the mixed Li–Pb layer, wherein there are two parallel planes within the same Li–Pb layer stacked on top of one another. The stacking behavior is shown in Figure 2. The planes occupied by *d* also stack on top of one another, but unlike occupation in the mixed Li–Pb layer, this is due to the stacking of the pure Li layers in Li_8_PbO_6_. The *b* and *c* sites are found within the pure O layer. The arrangement of the interstitial sites across each layer is shown in Figure 3.

**Figure 4:**
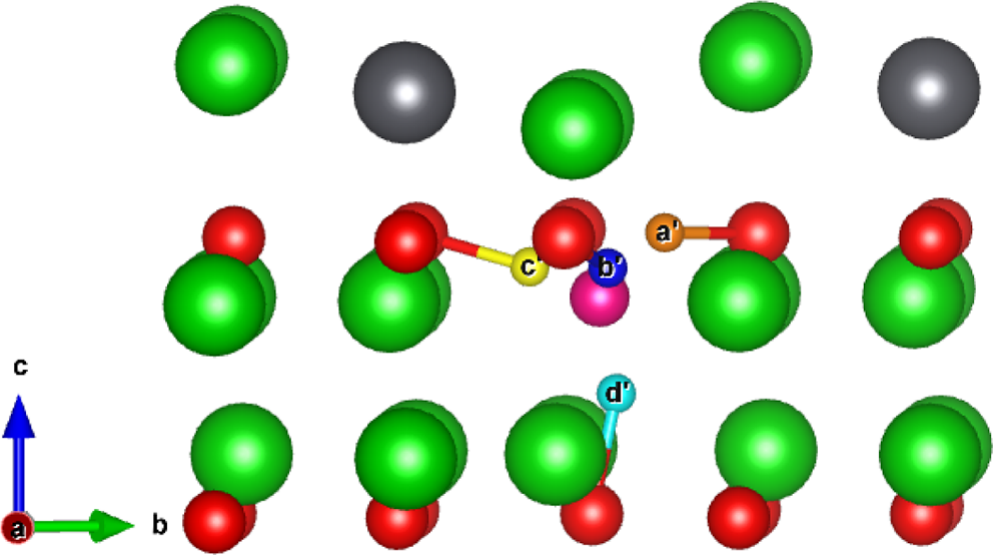
Possible tritium hydroxyl sites within the V_Li1_ defect. Orange, blue, yellow, and teal ions represent the *a’* - *d’* sites, respectively.

**Figure 5:**
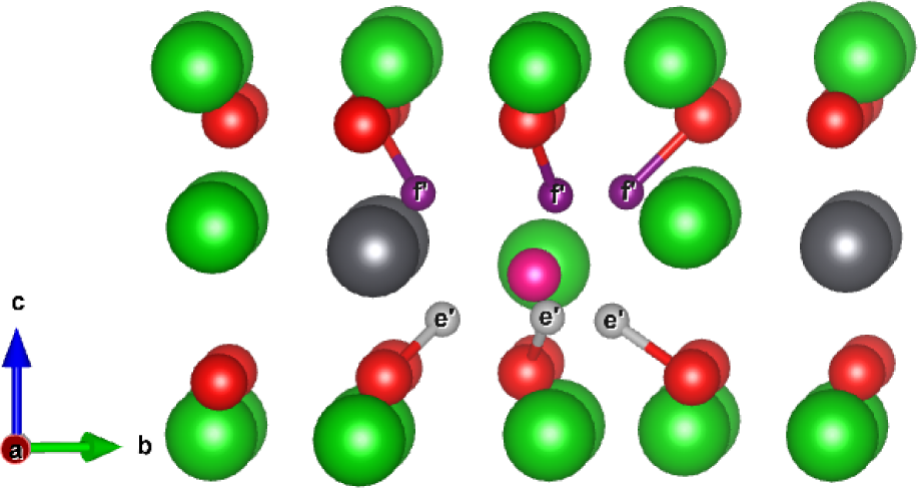
Possible tritium hydroxyl sites within the V_Li2_ defect. Dark red and purple ions represent the *e’* - *f’* sites, respectively.

Introduction of T into a V_Li_ defect by repeating the same method of allowing T to relax from the midpoint between the lithium vacancy and the neighboring oxygen ions to form a {:}^×^ defect cluster, as observed in Li_2_TiO_3_,^39^ causes the hydroxyl to point almost directly toward the lithium vacancy. Surrounding the V_Li2_ defect, there are now 2 symmetrically distinct sites for the tritium to occupy. As was the case for interstitial V_Li1_, there remain four unique sites surrounding the Li1 site. For clarity, the four symmetrically distinct sites found in V_Li1_ corresponding to interstitial sites a-*d* shall be labeled *a’*-*d’*. The two new sites found surrounding V_Li2_ shall be labeled with *e’* and *f’*. Illustrations of the {:} defect cluster at the Li1 and Li2 sites are shown in Figures 4 and 5.

Formation energies for all tritium-accommodating defects are presented in Table 1. A comparison is drawn on the incorporation of temperature effects into the chemical potentials for the constituent compounds Li_2_O, PbO_2_ as well as Li_8_PbO_6_ used in calculating the defect formation energies, similar to what was done in previous work.^28^

**Table 1:** Formation Energies of Tritium Accommodating Defects in Li_8_PbO_6_ under Li_2_O-Rich Conditions at the VBM^a^

	Defect	Site	E_*f*_ (eV)	E_*f*_(T) (eV)
1		*a*	2.85	3.04
2		*b*	3.11	3.30
3		*c*	3.05	3.24
4		*d*	3.34	3.53
5		*a*	–0.30	–0.11
6		*b*	0.06	0.25
7		*c*	–0.07	0.12
8		*d*	0.10	0.29
9		*a*′	1.27	1.25
10		*b*′	1.17	1.15
11		*c*′	1.12	1.09
12		*d*′	1.43	1.40
13		*e*′	1.46	1.44
14		*f*′	1.57	1.55
15		*a*′	0.15	0.13
16		*b*′	0.22	0.19
17		*c*′	0.31	0.28
18		*d*	0.43	0.41
19		*e′*	0.49	0.47
20		*f*′	0.60	0.58
21			9.17	9.32
22			6.05	6.20
23			3.01	3.16
24			8.56	8.71

a *T* = 1000 K, OPP = 0.2 atm, and c_T_ = 10^–5^.

**Figure 6:**
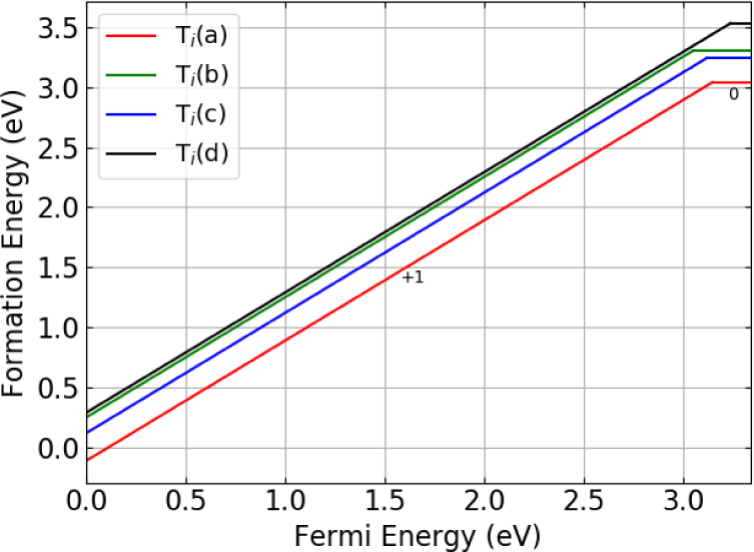
Defect formation energies for the tritium interstitial in Li_8_PbO_6_ as a function of the Fermi energy.

In the interstitial case, regardless of charge state, tritium prefers to occupy the mixed Li–Pb plane at site *a*, and least prefers site *d* in between the pure Li planes with a relative formation energy 0.49 eV greater than that of site a. The energies for the two sites in the oxygen plane (*b* and *c*) are predicted to have very similar energies. An illustration of the formation energies for the charge states of the tritium interstitial defects relative to the Fermi level within the band gap is given in Figure 6, and it demonstrates the preference for tritium to occupy the +1 charge state, rather than the charge-neutral state.

**Figure 7:**
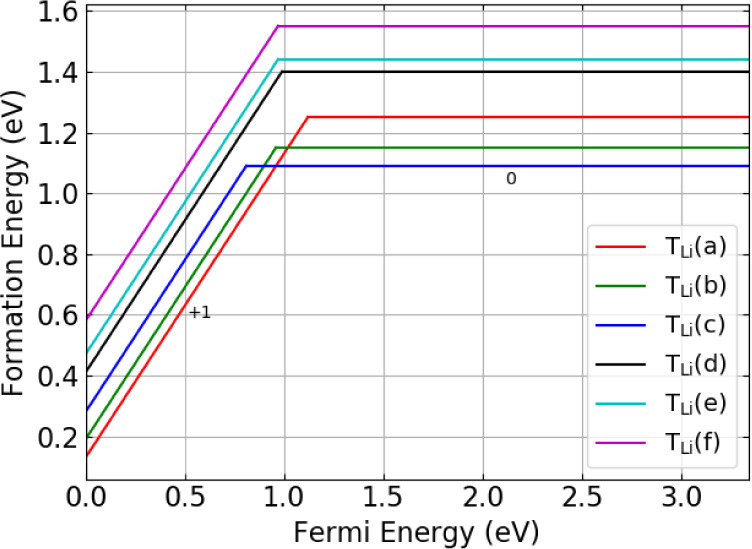
Defect formation energies for the {T:V_Li_} defect cluster in Li_8_PbO_6_ as a function of the Fermi energy.

For the {T:V_Li_} defect clusters (illustrated in Figure 7), the overall charge state of the cluster ultimately determines the preferred occupation of the defect in the material. In the charge-neutral case, the *c’* site is the preferred occupation state for the cluster situated on the Li1 site and for the Li2 site, *e’* is preferred over *f’*. When a +1 charge is introduced, the preference for the cluster around the Li1 site changes to the *a’* configuration, which is the lowest energy configuration overall.

**Figure 8:**
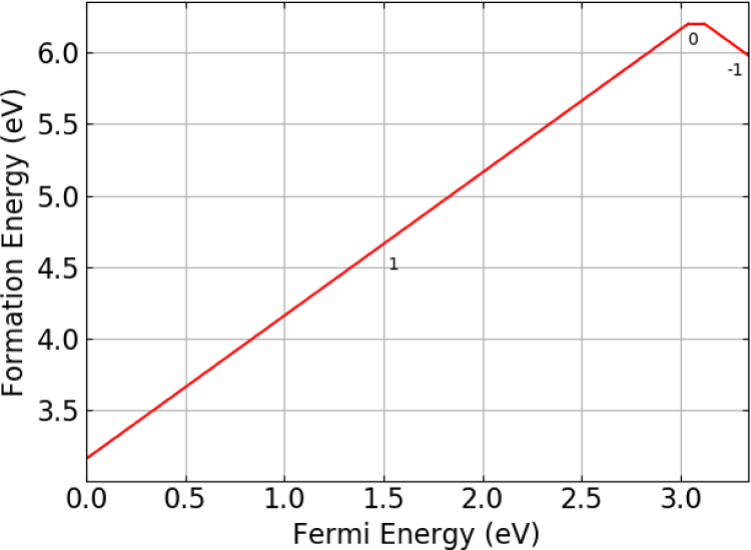
Defect formation energies for the {T:V_O_} defect cluster in Li_8_PbO_6_ as a function of the Fermi energy.

Figure 8 shows the formation energy of the T_O_ defect as a function of the Fermi level. The figure suggests that the dominant charge state for the defect is +1, where the tritium exists as a hydride with *a* −1 charge sitting on the doubly positively charged oxygen vacancy. This observation is identical to what is seen in Li_2_TiO_3_.^39^

The discrepancy in formation energies predicted between E_*f*_ and E_*f*_(T) is effectively negligible, with a roughly 0.02 eV difference in the substitutional cases, and in the interstitial case, there is a 0.19 eV discrepancy, much smaller than what was previously seen in the intrinsic case.^28^ However, this is to be expected as the chemical potentials of Li and Pb derived from Gibbs energies of Li_2_O and PbO_2_ having no impact on the chemical potential of hydrogen used in DefAP, as well as the fixing of the hydrogen chemical potential to half the DFT lattice energy of a H_2_ dimer as opposed to using the Gibbs energy.

### Tritium Accommodation

Similar to previous work studying the intrinsic defect chemistry of Li_8_PbO_6_ we use the DefAP^40^ code to predict defect concentrations under different environmental conditions. Defect formation energies for intrinsic defects are taken from previous work. In construction of these plots, the tritium concentration *c*_T_ is set to 10^–5^ and 10^–7^ per formula unit. The oxygen partial pressure (OPP) is fixed to 0.2 atm to represent typical atmospheric conditions, as operating conditions are currently unknown. To assess the influence of the OPP on the accommodation mechanisms of tritium, the OPP is also reduced to 10^–20^ atm and compared with standard atmospheric conditions. The influence of OPP on intrinsic defects is also showcased in our previous work.^28^

**Figure 9:**
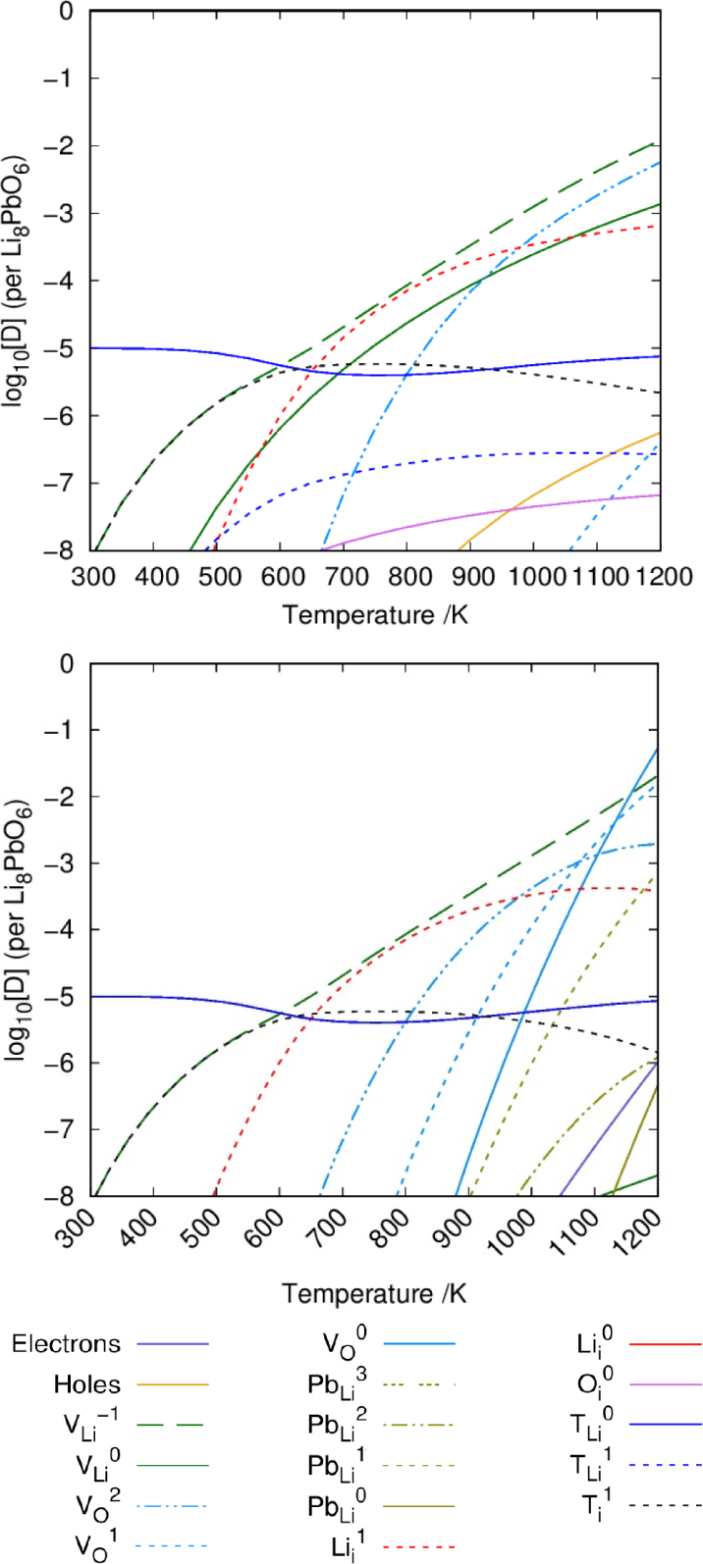
Defect chemistry of tritium accommodated Li_8_PbO_6_ under Li_2_O-rich conditions. The top figure uses an OPP of 0.2 atm, whereas the bottom figure uses 10^–20^ atm. In both instances, *c*_T_ = 10^–5^ per formula unit.

Li_8_PbO_6_ is expected to be stable under Li_2_O-rich conditions according to the Li_2_O-PbO_2_ phase diagram described in previous work,^28^ and thus only tritium accommodation under these conditions are considered here. In Li_8_PbO_6_, tritium is accommodated primarily as a substitutional defect on the V_Li_ vacancy site as a charge-neutral {:} cluster (Figure 9). For moderate temperatures in the range of 600–900 K, the charged interstitial becomes the dominant tritium accommodating defect in the crystal. It should be noted that even under very small oxygen partial pressures, tritium prefers to accommodate on the lithium vacancy site and as an interstitial rather than on the oxygen vacancy. Aside from the intrinsically higher concentration of oxygen vacancy defects when reducing the oxygen partial pressure, the similarity between the Brouwer diagrams shown in Figure 9 suggests the oxygen environment has little influence on the accommodation mechanisms of tritium in Li_8_PbO_6_.

**Figure 10:**
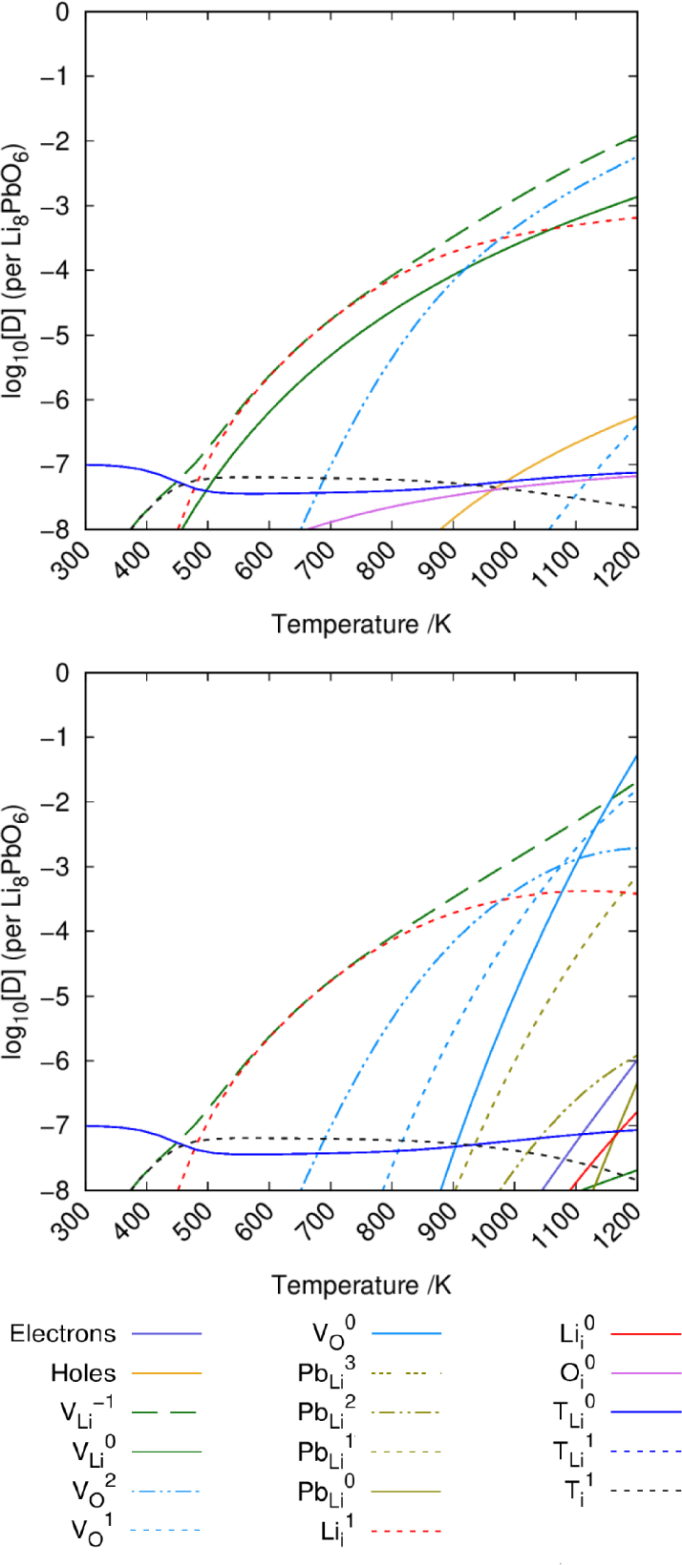
Defect chemistry of tritium accommodated Li_8_PbO_6_ under Li_2_O-rich conditions with a reduced tritium concentration. The top figure uses an OPP of 0.2 atm, whereas the bottom figure uses 10^–20^ atm. In both instances, *c*_T_ = 10^–7^ per formula unit.

Reducing the total concentration of tritium, the accommodation mechanism for tritium in Li_8_PbO_6_ (given by Figure 10) remains roughly the same, although the temperature range for which  is the dominant mechanism expands to lower temperatures, with an approximate range of 460–900 K, suggesting a general preference for tritium to accommodate as an interstitial until the crystal is highly saturated.

**Figure 11:**
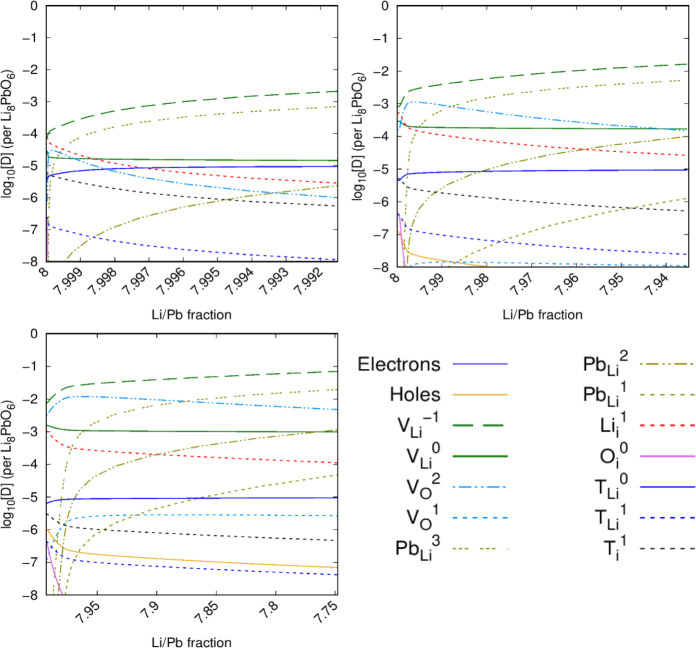
Tritium accommodation mechanism in Li_8_PbO_6_ as a function of lithium burn-up (Li:Pb ratio). OPP = 0.2 atm. Top left: *T* = 800 K. Top right: *T* = 1000 K. Bottom left: *T* = 1200 K. *c*_T_ = 10^–5^ per formula unit.

Similar to our previous work on the intrinsic chemistry of Li_8_PbO_6_,^28^ the defect chemistry during Li burn-up has been predicted with the inclusion of tritium in Figure 11 under various temperatures. Figure 11 suggests that at the start of its operational life, where the Li/Pb fraction is expected to be 8, there is a small region where the tritium interstitial is the dominant mode of tritium accommodation at low temperature. However, at high temperature and also at low temperature, once some lithium has undergone transmutation, the  defect dominates.

The Li/Pb ratio in Li_8_PbO_6_ is not expected to drop below 7.75 for temperatures up to 1200 K, although it should be warned that extending the dilute limit energetics for large deviations of the Li/Pb ratio from the dilute limit may be unrepresentative of the true defect chemistry, and care should be taken to consider simulations of the exact nonstoichiometry for significant changes in the cation ratio. Overall, these results suggest that under all of the conditions studied, tritium is accommodated as either an interstitial or bound to a lithium vacancy defect; therefore, only diffusion mediated by these defects will be considered in the remainder of this work.

### V_Li_ Migration

The lithium vacancy is predicted to be the dominant intrinsic defect in Li_8_PbO_6_, and therefore, it is important to understand how it behaves in the crystal as well as its interaction with tritium.

**Figure 12:**
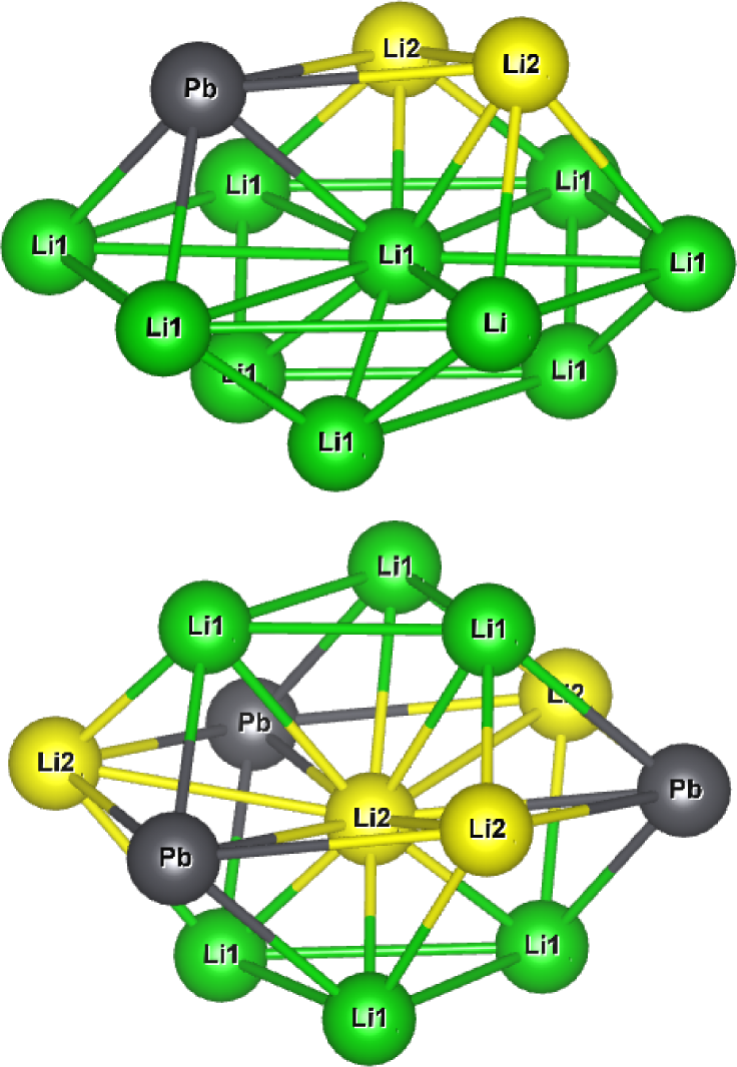
Neighboring cation environment for the Li1 and Li2 lithium sites.

The neighboring cation environments for Li1 and Li2 are illustrated in Figure 12. Surrounding the Li1 site are 12 neighboring cations, 11 of which are other lithium ions: 6 in the same pure lithium plane, 3 in the neighboring lithium plane, and 2 in the mixed Li–Pb plane. Although due to symmetry, there are only 6 distinct nearest neighbor pathways for the lithium vacancy to migrate: 3 within the same pure-Li plane; 1 into the neighboring pure-Li plane; and 2 into the mixed Li–Pb plane. Li2 also has 12 neighboring cations, although 3 of these are lead ions in the same mixed Li–Pb plane, resulting in 9 neighboring lithium ions. Of which, there are only 3 symmetrically distinct pathways: 1 within the same mixed Li–Pb plane, and 1 in each of the neighboring pure-Li planes above and below. Activation energies for these processes are listed in Table 2.

**Table 2:** Activation Energies for the Migration of  Lithium Vacancy Defects

Plane	Pathway	*d* (Å)	Forward (eV)	Reverse (eV)
Above Li–Pb	V_Li1_ → V_Li2_	2.784	0.49	0.14
	V_Li1_ → V_Li2_	2.486	0.44	0.10
Same Li	V_Li1_ → V_Li1_	2.932	0.52	0.52
	V_Li1_ → V_Li1_	3.499	1.61	1.61
	V_Li1_ → V_Li1_	3.267	0.84	0.84
Below Li	V_Li1_ → V_Li1_	2.374	0.33	0.33
Above Li	V_Li2_ → V_Li1_	2.784	0.14	0.49
Same Li–Pb	V_Li2_ → V_Li2_	3.278	0.72	0.72
Below Li	V_Li2_ → V_Li1_	2.486	0.10	0.44

It was found that regardless of which charge state was chosen for the V_Li_ defect, there is virtually no difference in activation energy, with the greatest difference in activation energies occurring in the transition between the V_Li2_ and V_Li1_ states with the charge-neutral state possessing an activation energy 11 meV greater than that for the charged defect migration. Due to this, we assume the barriers for diffusion of both charge states for the V_Li_ defect to be the same.

The lowest migration barriers found are those for V_Li2_ migrating to the V_Li1_ site, with barriers of 0.10 and 0.14 eV. This can be explained by the lower formation energy possessed by V_Li1_ compared to the V_Li2_ defect^19^ caused by the lower number of Li–O bonds the Li^1+^ ion has to break to be removed from the Li1 site.

**Figure 13:**
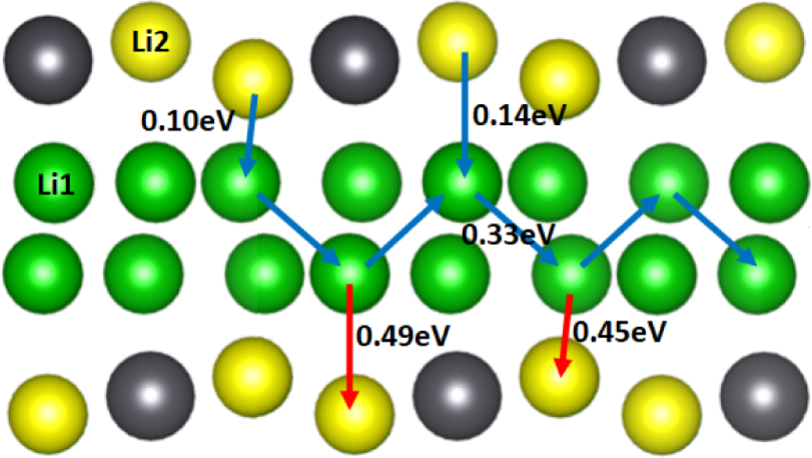
Migration pathway for the  defect. Green and yellow ions are the Li1 and Li2 sites, respectively. Oxygen ions are not shown to enable easier visualization.

Once the lithium vacancy is in a pure-Li plane, the optimal pathway for it to migrate is to the neighboring pure-Li plane with an activation barrier of 0.33 eV. The optimal migration pathway for the lithium vacancy defect is to hop between and migrate through the pure-Li layers in the *xy*-plane until a grain boundary is reached, rather than migrate to a mixed Li–Pb plane which has migration barriers of 0.44 and 0.49 eV. An illustration of the lithium migration is shown in Figure 13.

Therefore, the overall barrier for migration in the *xy*-plane is 0.33 eV, and diffusion in the *z*-direction is 0.44 eV.

### T Interstitial Migration

As illustrated in Figures 2 and 3 in section Tritium Defect Formation Energies, there are 4 unique interstitial sites the tritium can occupy: site *a* in the mixed Li–Pb plane; two sites *b* and *c* within the oxygen plane which lies between the pure Li and mixed Li–Pb planes; and finally site *d* which exists in the pure Li plane. In total, 23 unique pathways for tritium interstitial migration were found and considered. The pathways and their respective activation energies are listed in Table 3.

**Table 3:** Activation Energies for the Migration of  Tritium Interstitial Defects^a^

Initial Plane	Final Plane	Pathway	*d* (Å)	Forward (eV)	Reverse (eV)
Li–Pb	Li–Pb	*a* → *a*	2.729	0.56	0.56
		*a* → *a*	2.943	0.77	0.77
		*a* → *a̅*	1.199	0.29	0.29
		*a* → a̅	2.630	0.88	0.88
	Oxygen	*a* → *b*	2.042	0.74	0.47
		*a* → *b*	1.397	0.26	0
		*a* → *c*	1.902	0.56	0.34
		*a* → *c*	1.355	0.22	0
Oxygen	Oxygen	*b* → *b*	1.808	0.48	0.48
		*c* → *b*	2.555	0.73	0.67
		*c* → *b*	1.992	1.89	1.84
		*c* → *c*	1.486	0.35	0.35
Pure-Li	Li–Pb	*d* → *a*	2.683	0.46	0.90
	Oxygen	*d* → *b*	2.043	0.29	0.45
		*d* → *b*	2.226	0.47	0.63
		*d̅* → b	1.580	0.32	0.48
		*d* → *c*	2.378	0.46	0.68
		*d* → *c*	2.293	0.47	0.69
		*d̅* → c	1.304	0	0.20
	Pure-Li	*d* → *d*	2.528	0.50	0.50
		*d* → *d*	3.247	0.13	0.13
		*d* → *d̅*	2.065	0.45	0.45
		*d* → *d̅*	2.364	0.27	0.27

a Accented sites represent interstitials which lie on a neighboring interstitial plane.

**Figure 14:**
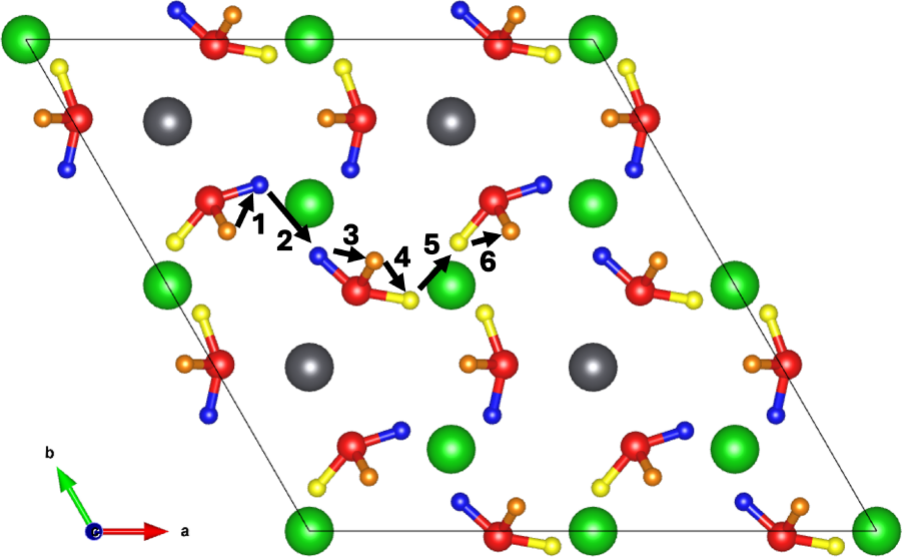
Optimal migration pathway of the tritium interstitial through the combined pure and mixed Li–Pb planes. Numbers represent the order of the migratory hops along the *a → b → b → a → c → c → a* pathway.

Given the high number of options available to the tritium interstitial, we discuss only the most favorable pathways along each axis. Considering first the migration of the tritium interstitial within the mixed Li–Pb, oxygen, and pure-Li planes separately, migration exclusively through the oxygen plane is found to have the highest potential barrier, with a pathway of *c → c → b → b → c*, and a total barrier of 0.73 eV for the entire migratory process. If instead of migrating directly between sites *b* and *c* (which share the same T-O hydroxyl bond), an intermediary step is placed by allowing the hydroxyl to rotate into the mixed Li–Pb plane to site *a* such that *b → a → c* (and vice versa), the activation energy of the migration along the oxygen plane reduces to 0.74 eV and a full pathway of *a → b → b → a → c → c → a*, as illustrated in Figure 14.

**Figure 15:**
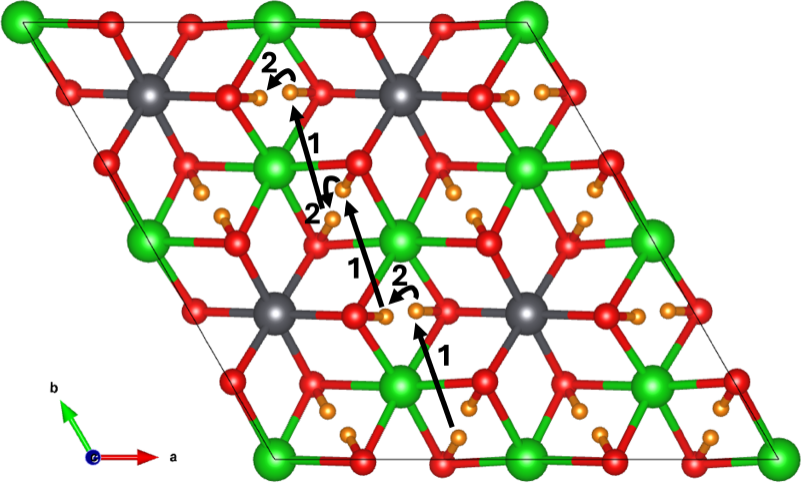
Migration pathway of the tritium interstitial phase within the mixed Li–Pb plane. Numbers represent the order of unique hops along the *a → a → a̅ → a̅ → a*.

Migrating through the mixed Li–Pb plane results in a lower migration barrier than for the case of migration through the oxygen plane. As seen in Figure 2, within the mixed Li–Pb plane, there exist two stacked planes the tritium interstitial can occupy (site *a*). The most efficient pathway is for the tritium to hop between the two subplanes once for every occurrence the tritium migrates within the same subplane, i.e., *a → a → a̅ → a̅ → a*, with the bar used to distinguish between interstitial sites on opposite subplanes. With such a pathway, the total activation energy for the entire migration is 0.56 eV, and an illustrative example is given in Figure 15.

**Figure 16:**
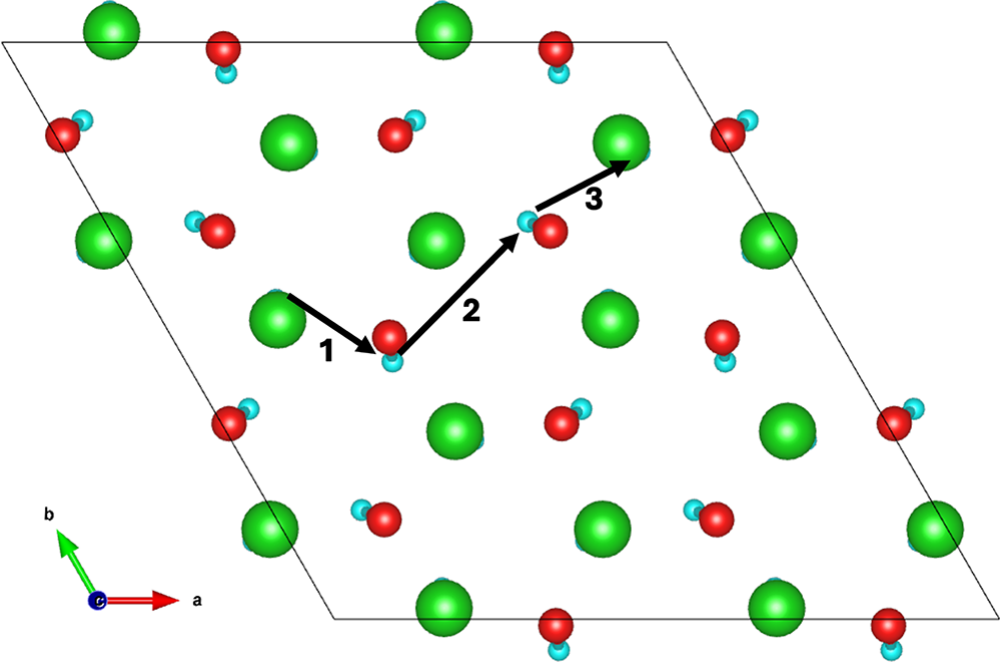
Migration pathway of the tritium interstitial through the pure-Li subplane. Numbers represent the order of the migratory hops along the *d → d̅ → d̅ → d* pathway.

Migration exclusively within the pure-Li plane of the tritium interstitial defect appears much more favorable. Much like the case of migration through the mixed Li–Pb plane, there are two subplanes the tritium interstitial occupies within the pure-Li plane stacked on top of one another, and the tritium utilizes both planes in order to migrate. Relative to the previous pathways considered, migration anywhere through the pure-Li plane yields a lower activation barrier, with a maximum barrier of 0.50 eV. The pathway yielding the lowest activation barrier is that of the migration *d → d̅ → d̅ → d*, with an overall barrier of 0.27 eV. Such a low barrier is due to the presence of a semistable site for the tritium interstitial along the subpath *d → d* yielding a barrier of 0.13 eV. An illustration of the pathway is given by Figure 16. It is expected that the majority of tritium interstitial migration will occur along the pure-Li plane, as the tritium is predicted to vacate the mixed Li–Pb and oxygen planes via *c → a*. The barrier for this pathway is very low and is effectively 0 eV. However, there must be a barrier, as the final position is a stable site. This is similar to the observations made by Shi et al. for Li_2_TiO_3_.

**Figure 17:**
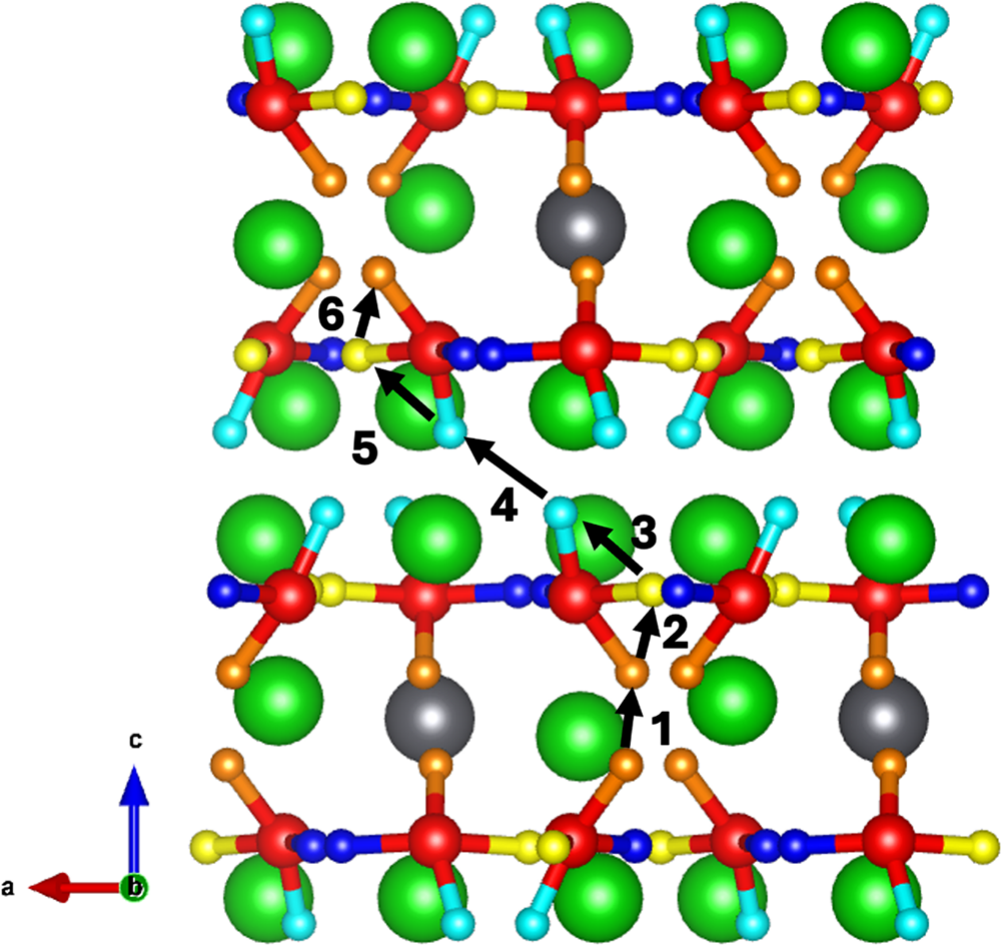
Migration pathway of the tritium interstitial pillar vertically through the crystal. Numbers represent the order of the migratory hops along the *a̅ → a → c → d̅ → d → c̅ → a̅* pathway.

In the vertical direction perpendicular to the mixed Li–Pb and pure-Li planes, the optimal pathway is predicted to have an activation energy of 0.69 eV. Beginning in the mixed Li–Pb plane from site *a*, the optimal pathway for the tritium interstitial to take is predicted to be *a̅ → a → c → d̅ → d → c̅ → a̅* (see Figure 17).

Overall, the pathway containing the semistable state through the pure-Li plane along the *xy*-axes via the process *d → d̅→ d̅ → d* is the most preferred, with an energy barrier of 0.27 eV. The activation energy for migration is anisotropic, as is expected from the intrinsically anisotropic crystal structure of Li_8_PbO_6_, with a barrier along the *z*-axis of 0.69 eV. Migration barriers for the pathways illustrated in Figures 14–17 are shown in Figures S1–S5 in the Supporting Information.

### {:}^0^ Internal Migration

Figures 4 and 5 illustrate the possible hydroxyl positions for the tritium to form within the V_Li1_ and V_Li2_ environments, where due to Coulombic attraction, the hydroxyls have reorientated such that the tritium is directed toward the vacancy. It is expected the tritium trapped within these {:} defect clusters will reorient themselves prior to escaping the trapping site. Therefore, in Table 4, activation energies for the internal migration of tritium within these defect clusters are presented.

**Table 4:** Activation Energies for the Internal Migration of Tritium within the V_Li1_ and V_Li2_ Vacancy Defects

			Barrier {:}^0^		Barrier {:}^+1^	
Vacancy site	Pathway	*d* (Å)	Forward (eV)	Reverse (eV)	Forward (eV)	Reverse (eV)
V_Li1_	a′ → b′	1.319	0.49	0.43	0.56	0.50
	a′ → c′	1.311	0.54	0.38	0.60	0.46
	a′ → d′	1.599	0.78	0.47	0.84	0.56
	b′ → c′	1.566	0.76	0.66	0.81	0.72
	b′ → d′	1.395	0.67	0.42	0.74	0.52
	c′ → d′	1.542	0.77	0.62	0.83	0.69
V_Li2_	e′ → e′	1.757	0.71	0.71	0.78	0.78
	e′ → f′	2.067	0.58	0.47	0.65	0.54
	e′ → f′	2.375	1.06	0.95	1.12	1.01
	f′ → f′	2.153	1.01	1.01	1.08	1.08

From Table 4, it is clear introducing a +1 charge state does not have a significant impact on the activation energies, with differences ranging from 0.05–0.07 eV. We can see from the table that there are 6 possible reorientations around the V_Li1_ site and only 4 surrounding V_Li2_ due to symmetry.

First observing V_Li1_, *a’* is found to be the most favorable position for the other hydroxyl groups to reorient toward, and *d’* the least favorable, with the highest barrier for reorientation toward *a’* of 0.47 eV and the lowest barrier for reorientation toward *d’* of 0.67 eV in the {:}^0^ charge-neutral defect cluster case, which is in agreement with the formation energies of the T^+1^ defects seen in Table 1.

For reorientation around the V_Li2_ defect, the hydroxyl groups are predicted to migrate toward the *e’* position, rather than *f’*, with the lowest barrier for migration being 0.47 eV from *f’* to *e’*, as expected from the formation energies presented in Table 1. Interestingly, the second lowest energy barrier is the reverse process for the same pathway, with 0.58 eV, so it is expected the hydroxyl will primarily only swap positions along this pathway, rather than migrate to a symmetric hydroxyl site or take the alternative *e’* to *f’* pathway.

### Tritium Escape

For tritium to escape from the lithium vacancy cluster, it is expected to reorient itself around the oxygen it is bound to, until it is directed away from the lithium vacancy site, where the tritium is far enough away from the lithium vacancy defect to be treated as an interstitial. In this section, we measure the activation energy barriers for rotation of the hydroxyl groups away from the {:}^0^ defect cluster, wherein tritium can then be treated as an interstitial.

**Figure 18:**
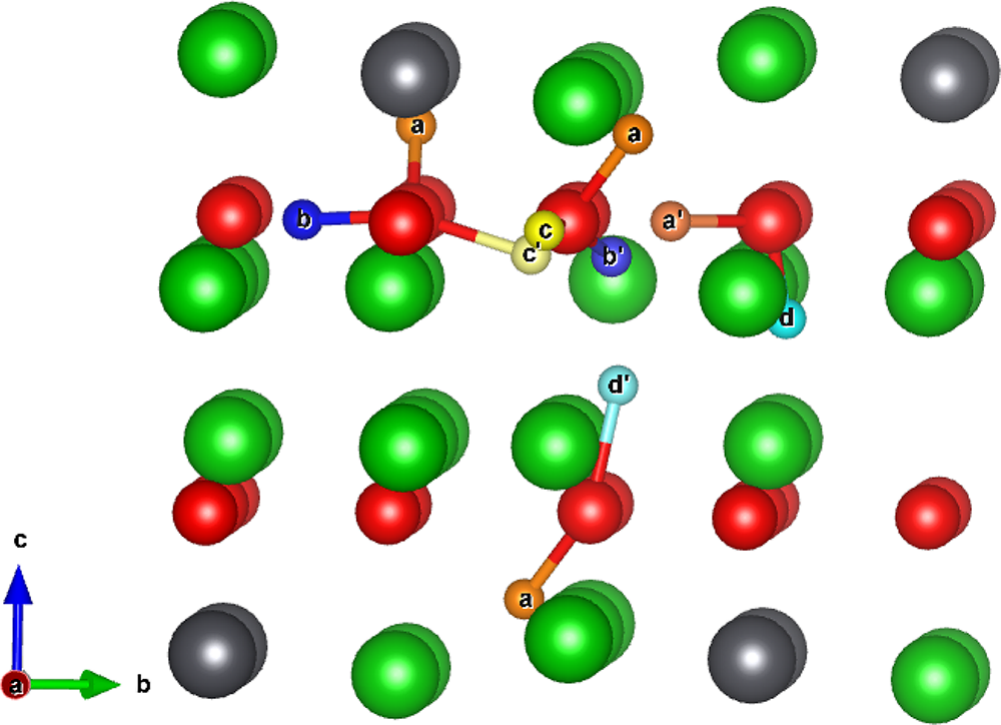
Reorientation pathways for escape from the {T^+1^:} defect.

**Figure 19:**
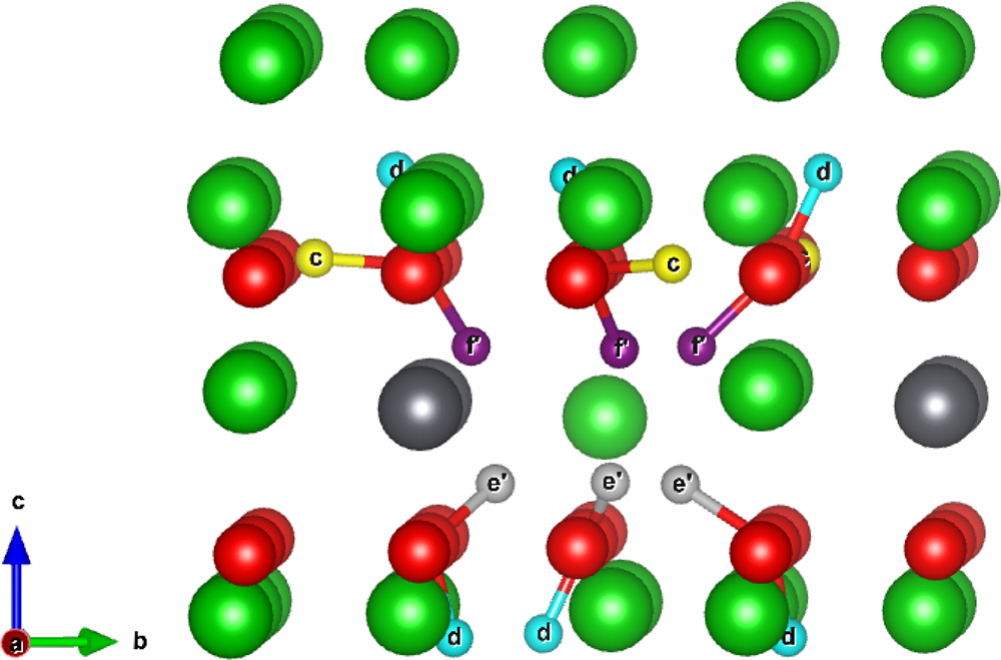
Reorientation pathways for escape from the {T^+1^:} defect.

The final positions for reorientation are assumed to be the previously considered interstitial sites *a*-*d*, and beginning from each initial position *a’*-*f’*, each initial site has 3 potential final sites. For example, *a’* may reorient itself into the *b*, *c*, or *d* positions. The final positions were first allowed to structurally relax, and following relaxation of the 18 potential final sites for the tritium (3 for each initial site), only 9 were found to be stable at the interstitial positions. The rest either migrated to a more stable position or reoriented back into the defect cluster. The activation energies for tritium escape via reorientation of the hydroxyl around a bound oxygen are shown in Table 5, and illustrations of the possible reorientation pathways away from the V_Li1_ and V_Li2_ trapping sites are shown in Figures 18 and 19 respectively.

**Table 5:** Barriers for Tritium Escape from the {:} Defect Cluster^a^

Vacancy site	Escape pathway	*d* (Å)	Forward (eV)	Reverse (eV)
V_Li1_	*a*′ → *d*	1.676	>5*	>5*
	b′ → *a*	1.825	0.43	0.13
	*b*′ → *c*	2.210	0.82	0.37
	*c*′ → *a*	1.804	0.39	0.10
	*c*′ → *b*	2.132	0.47	0
	*d*′ → *a*	2.136	5.02*	5.01*
V_Li2_	*e*′ → *d*	1.762	0.91	0.20
	*f*′ → *c*	1.709	0.42	0
	*f*′ → *d*	2.022	4.36*	3.69*

a An asterisk indicates an implausible reaction pathway due to a failure to converge.

Escape from the V_Li1_ trapping site showed reasonably low activation energies for reorientation between 0.43 and 0.82 eV. As expected, the reverse process for each migration is significantly lower, due to the attraction of the T^+1^ interstitial to the oppositely charged  defect. The migration pathways from *a′ → d* and *d′ → a* were found to have very high activation energies, suggesting these mechanisms are unlikely to contribute to tritium diffusion. It is much more likely the pathways for escape from *a’* and *d’* to first include a repositioning to *b’* or *c’* before attempting to escape the defect cluster. For *a’*, the optimal pathway for escape is to reorient to *b’* with a barrier of 0.47 eV, followed by escape to *a* outside the defect cluster with a barrier of 0.43 eV. The optimal pathway for escape from the *d’* site also requires a reorientation to *b’* within the cluster first, with an activation energy for the process of reorientation being 0.42 eV. For the lowest escape barrier considered of *c′ → a*, it is expected once the tritium is at the *a* site it will migrate along the *a → a → a̅ → a̅ → a* pathway, with a total activation energy of 0.85 eV.

Only three unique pathways were found to escape from the V_Li2_ site. Once again, much like we observed in the case of escape from the V_Li1_ trapping site, the *f′ → d* pathway possesses a very high activation energy. As the activation energy for migration from *f′ → d* is so high, it is likely tritium at the *f’* site will migrate exclusively via *f′ → c*, with an activation energy of 0.42 eV. As the activation energy for diffusion along the *c → a* pathway was previously measured to be 0 eV from Table 3, tritium is expected to migrate from *f’* to *c*, followed by a hop from *c* to *a*, where it escapes along the *a → a → a̅ → a̅ → a* pathway once again, resulting in a final pathway of *f′ → c → a → a → a̅ → a̅ → a* and an activation energy of 0.76 eV. The migration barriers to escape entirely from the crystal from *c’* and *f’* trapping sites are given in Figures S6 and S7 in the Supporting Information.

Overall, in comparison with other materials such as Li_2_TiO_3_, the barriers for escape from the {:} trapping site overall are notably lower and the lowest pathways for escape from the V_Li1_ and V_Li2_ trapping sites being 0.39 and 0.42 eV, respectively, compared to that for Li_2_TiO_3_, which sees the lowest barriers possessing activation energies of 0.55 and 0.66 eV for Li_2_TiO_3_.^15^ Comparing escape barriers from the crystal as a whole, Li_8_PbO_6_ exhibits significantly lower activation barriers in comparison with Li_2_TiO_3_, with barriers of 0.76–0.85 eV in the Li_8_PbO_6_ case, compared to that of Li_2_TiO_3_ which is predicted to have a minimum barrier of 1.05 eV, which may culminate in a higher tritium release rate for Li_8_PbO_6_ overall as tritium is less bound to the trapping sites.

### {:} Cluster Migration

**Figure 20:**
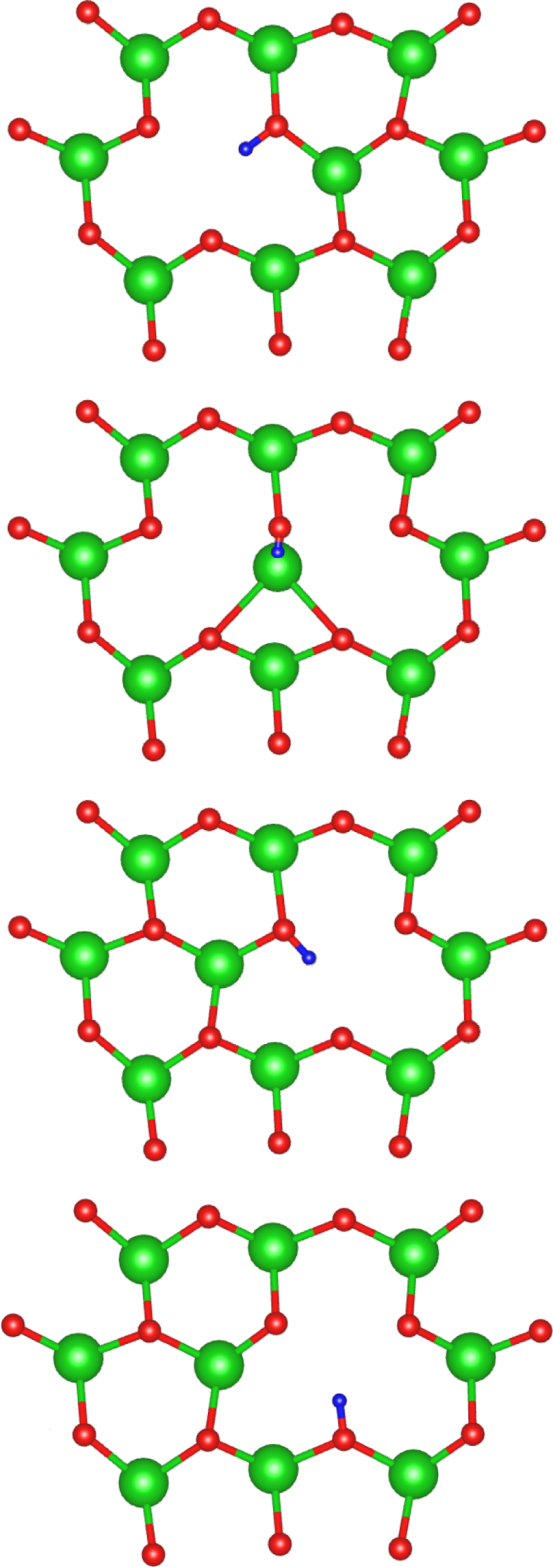
Illustration of migration of a {:} defect cluster. In this example, the cluster migrates along the , T(*a’*) → , T(*b’*) pathway, before the T-O bond is broken and migrates via the T(*b’*) → T(*a’*) pathway internally within the new lithium vacancy defect.

In this section, instead of considering the migration of the tritium alone, we instead consider the migration of the whole defect cluster, examining if simultaneous migration with the lithium vacancy defect helps to aid in the migration of tritium. The process for cluster migration is broken down into two stages: The first is a collaborative diffusion where a tritium hydroxyl simultaneously reorients around the bound oxygen as the lithium vacancy it is trapped in also migrates. The second is the breaking of the hydroxyl bond, such that the tritium internally diffuses within the newly positioned lithium vacancy. An illustrative example of the complete migration process for a defect cluster is shown in Figure 20.

A total of 11 possible migration pathways for the collaborative diffusion mechanism were found. Six beginning and ending at Li1 lithium sites exclusively ( → ), 4 of which change lithium vacancy sites between Li1 and Li2, and only one site where the lithium vacancy site begins and ends at Li2. Energy barriers for the collaborative diffusion pathways are listed in Table 6.

**Table 6:** Migration Barriers for the {:} Defect Cluster

pathway	pathway	*d*_T_ (Å)	(Å)	Forward (eV)	Reverse (eV)
	a′ → b′	1.660	2.932	1.08	1.03
	b′ → c′	2.202	3.499	2.30	2.19
	c′ → a′	1.778	3.267	3.73	3.88
	a′ → d′	1.712	2.374	2.20	1.89
	b′ → d′	1.475	2.374	1.20	0.94
	c′ → d′	1.264	2.374	0.85	0.70
	e′ → f′	1.361	3.278	2.61	2.50
	e′ → a′	0.838	2.486	0.78	1.12
	e′ → c′	1.010	2.486	0.50	0.69
	f′ → a′	0.961	2.784	0.73	1.18
	f′ → b′	1.499	2.784	0.26	0.66

The lowest migration barriers for the collaborative diffusion mechanism were found to be those along the  →  pathway, with barriers ranging from 0.26 to 0.78 eV. Due to such low barriers, it is evident there is indeed collaborative diffusion occurring between the migration of the lithium vacancy and tritium interstitial sites in Li_8_PbO_6_. Between Li1 sites, the lowest barrier was found to be 0.70 eV, and the highest 3.88 eV, although half of all barriers are relatively small, with sizes at or smaller than 1.25 eV. Considering the most energetically favorable collaborative diffusion process ( → ) followed by an internal migration within the new lithium vacancy site, as described in the {:}^0^ Internal Migration subsection, the total barrier for diffusion ranges between 0.67–1.18 eV. Alternatively, if the  →  pathway is considered first, the total barrier for diffusion is between 0.77–1.18 eV. Profiles of the potential energy surface are provided in the Supporting Information as Figures S8–S11.

Comparing with the escape pathways of tritium from the trapping sites and from the bulk crystal, it can be seen that the energies for tritium to escape from the lithium vacancy defect and move independently as an interstitial (0.76 eV) and for the two to diffuse together (0.67 eV) are very similar. Therefore, it is expected that the tritium will likely initially migrate collaboratively as part of a defect cluster before eventually escaping from the cluster and diffusing through the pure-Li plane before escaping the crystal entirely. Both of these values are remarkably similar to the 0.78 eV obtained from neutron-irradiated Li_8_PbO_6_ by Hayashi et al.^9^ As the sample used in this study had been irradiated with neutrons, it will also contain a large number of defects, including lithium vacancies, and so it is expected that the mechanisms studied here that consider the presence of these defects offer a better approximation to what is observed in the experiment.

## Conclusion

In this paper, we explored how tritium is accommodated and the mechanisms for tritium diffusion through bulk Li_8_PbO_6_ using Density Functional Theory. Tritium is expected to primarily occupy the lithium vacancy sites as a hydroxyl, forming a defect cluster, rather than as an isolated interstitial. The barriers for diffusion of tritium interstitials is anisotropic in nature, with a barrier of 0.27 eV in the *xy*-plane through the pure-Li planes and 0.69 eV vertically along the *z* direction. Similar anisotropy is also true for the lithium vacancy defect, which prefers to occupy and migrate through the pure-Li plane. For tritium to escape the crystal from the {:} defect cluster entirely there is a minimum barrier of 0.76–0.85 eV. The smallest activation energies for migration of the clusters as a whole, were between 0.67 and 1.18 eV, implying a collaborative diffusion mechanism between the tritium interstitials and lithium vacancy defects. Due to such low barriers, the aging of the blanket caused by lithium burn-up will have a smaller impact on tritium release compared to other materials.

## Supporting Information Available

Tritium interstitial reaction pathways (PDF)

## References

[ref1] Lucas L. L.; Unterweger M. P. Comprehensive review and critical evaluation of the half-life of tritium. J. Res. Natl. Inst. Stand. Technol 2000, 105, 541 10.6028/jres.105.043. 27551621 PMC4877155

[ref2] Brown R. M.; Grummitt W. E. The determination of tritium in natural waters. Can. J. Chem 1956, 34, 220–226. 10.1139/v56-033.

[ref3] Zheng S.; Todd T. Study of impacts on tritium breeding ratio of a fusion DEMO reactor. Fus. Eng. Des 2015, 98–99, 1915–1918. 10.1016/j.fusengdes.2015.06.171.

[ref4] Gilbert M.; Eade T.; Rey T.; Vale R.; Bachmann C.; Fischer U.; Taylor N. Waste implications from minor impurities in European DEMO materials. Nucl. Fusion 2019, 59, 076015 10.1088/1741-4326/ab154e.

[ref5] Giancarli L. M.; Abdou M.; Campbell D.; Chuyanov V.; Ahn M.; Enoeda M.; Pan C.; Poitevin Y.; Rajendra Kumar E.; Ricapito I.; Strebkov Y. Overview of the ITER TBM Program. Fusion Eng. Des 2012, 87 (5–6), 395–402. 10.1016/j.fusengdes.2011.11.005.

[ref6] Hernández F.; Pereslavtsev P.; Kang Q.; Norajitra P.; Kiss B.; Nádasi G.; Bitz O. A new HCPB breeding blanket design for DEMO: Evolution, rationale and preliminary performances. Fusion Eng. Des 2017, 124, 882–886. 10.1016/j.fusengdes.2017.02.008.

[ref7] Hernandez F. A.; Pereslavtsev P. First principles review of options for tritium breeder and neutron multiplier materials for breeding blankets in fusion reactors. Fus. Eng. Des 2018, 137, 243–256. 10.1016/j.fusengdes.2018.09.014.

[ref8] Palermo I.; Gómez-Ros J. M.; Veredas G.; Catalán J. P.; Ogando F.; Sanz J.; Sedano L. Preliminary neutronic assessment of a helium-cooled Li8PbO6 breeding blanket design for DEMO. Fusion Eng. Des 2012, 87 (2), 195–199. 10.1016/j.fusengdes.2011.12.016.

[ref9] Hayashi T.; Konishi S.; Okuno K. Tritium release behavior from neutron-irradiated Li_8_PbO_6_. J. Nucl. Mater 1990, 170, 60–65. 10.1016/0022-3115(90)90327-J.

[ref10] Kinjyo T.; Nishikawa M.; Enoeda M.; Fukada S. Tritium Diffusivity in Crystal Grain of Li_2_TiO_3_ and Tritium Release Behavior Under Several Purge Gas Conditions. Fusion Eng. Des 2008, 83 (4), 580–587. 10.1016/j.fusengdes.2007.11.011.

[ref11] Tanifuji T.; Yamaki D.; Nasu S.; Noda K. Tritium Release Behavior from Neutron-irradiated Li_2_TiO_3_ Single Crystal. J. Nucl. Mater 1998, 258–263, 543–548. 10.1016/S0022-3115(98)00103-2.

[ref12] Zhu D.; Oda T.; Shono Y.; Tanaka S. Release Behavior of Hydrogen Isotopes Thermally Sorbed in Li_2_TiO_3_ Single Crystal. J. Nucl. Mater 2013, 442, S437–S441. 10.1016/j.jnucmat.2013.02.062.

[ref13] Zhao L.; Long X.; Peng S.; Chen X.; Xiao C.; Ran G.; Li J. Tritium release in Li_4_SiO_4_ and Li_4.2_Si_0.8_Al_0.2_O_4_ ceramics. J. Nucl. Mater 2016, 482, 42–46. 10.1016/j.jnucmat.2016.10.009.

[ref14] Xiao C.; Gao X.; Kobayashi M.; Kawasaki K.; Uchimura H.; Toda K.; Kang C.; Chen; Wang H.; Peng S. Tritium release kinetics in lithium orthosilicate ceramic pebbles irradiated with low thermal-neutron fluence. J. Nucl. Mater 2013, 438, 46–50. 10.1016/j.jnucmat.2013.02.069.

[ref15] Goswami K. N.; Murphy S. T. Influence of Lithium Vacancy Defects on Tritium Diffusion in β-Li_2_TiO_3_. J. Phys. Chem. C 2020, 124, 12286–12294. 10.1021/acs.jpcc.0c02551. PMC7590524 33133328

[ref16] Kröger F.; Vink H.; Relations between the Concentrations of Imperfections in Crystalline Solids. Solid State Physics; Seitz F.; Turnbull D.; Eds.; Academic Press, 1956; Vol. 3, pp 307–435.

[ref17] De Souza R.; Harrington G. Revisiting point defects in ionic solids and semiconductors. Nat. Mater 2023, 22, 794–797. 10.1038/s41563-023-01583-4. 37386062

[ref18] Heyd J.; Scuseria G. E.; Ernzerhof M. Hybrid functionals based on a screened Coulomb potential. J. Chem. Phys 2003, 118, 8207 10.1063/1.1564060.

[ref19] Davies A. W.; Murphy S. T. Fundamental properties of octalithium plumbate ceramic breeder material. J. Nucl. Mater 2021, 552, 152982 10.1016/j.jnucmat.2021.152982.

[ref20] Colominas S.; Palermo I.; Abella J.; Gómez-Ros J. M.; Sanz J.; Sedano L. Octalithium plumbate as a breeding blanket ceramic: Neutronic performances, synthesis and partial characterization. Fus. Eng. Des 2012, 87, 482–485. 10.1016/j.fusengdes.2012.01.003.

[ref21] Davies A. W.; Murphy S. T. Thermodynamics and phase stability of Li_8_XO_6_ octalithium ceramic breeder materials (X = Pb, Ce, Ge, Zr, Sn). J. Phys.: condens. Matter 2022, 34, 355701 10.1088/1361-648X/ac762a. 35667375

[ref22] Togo A.; Tanaka I. First principles phonon calculations in materials science. Scr. Mater 2015, 108, 1–5. 10.1016/j.scriptamat.2015.07.021.

[ref23] Chase M. W. J. NIST-JANAF Thermochemical Tables, 4th ed.; NIST, 1998.

[ref24] Johnston H. L.; Bauer T. W. Low temperature heat capacities of inorganic solids, VII. Heat capacity and thermodynamic functions of Li_2_O. Thermodynamics of the Li_2_O-H_2_O system. J. Am. Chem. Soc 1951, 73, 1119–1122. 10.1021/ja01147a070.

[ref25] Glensk A.; Grabowski B.; Hickel T.; Neugebauer J. Understanding Anharmonicity in fcc Materials: From its Origin to *ab initio* Strategies beyond the Quasiharmonic Approximation. Phys. Rev. Lett 2015, 114, 195901 10.1103/PhysRevLett.114.195901. 26024182

[ref26] Finnis M. W.; Lozovoi A. Y.; Alavi A. The Oxidation of NiAl: What Can We Learn from Ab Initio Calculations?. Annu. Rev. Mater. Res 2005, 35, 167–207. 10.1146/annurev.matsci.35.101503.091652.

[ref27] Chase M. W. Jr.; Davies C. A.; Downey J. R.; Frurip D. J.; McDonald R. A.; Syverud A. N. NIST JANAF Thermochemical Tables 1985; NIST, 1986.

[ref28] Davies A. W.; Neilson W. D.; Bedford R. T.; Murphy S. T. The High Temperature Intrinsic Defect Chemistry of Li_8_PbO_6_ Ceramic Breeding Material. J. Phys. Chem. C 2023, 127, 22265–22276. 10.1021/acs.jpcc.3c04186. PMC10658618 38024197

[ref29] Linstrom P. J.; Mallard W. J. NIST Chemistry WebBook, NIST Standard Reference Database Number 69.

[ref30] Kresse G.; Furthmüller J. Efficient iterative schemes for ab initio total-energy calculations using a plane-wave basis set. Phys. Rev. B 1996, 54, 11169 10.1103/PhysRevB.54.11169. 9984901

[ref31] Kresse G.; Joubert D. From ultrasoft pseudopotentials to the projector augmented-wave method. Phys. Rev. B 1999, 59, 1758 10.1103/PhysRevB.59.1758.

[ref32] Perdew J. P.; Burke K.; Ernzerhof M. Generalized Gradient Approximation Made Simple. Phys. Rev. Lett 1996, 77 (18), 3865–3868. 10.1103/PhysRevLett.77.3865. 10062328

[ref33] Monkhorst H. J.; Pack J. D. Special points for Brillouin-zone integrations. Phys. Rev. B 1976, 13, 5188 10.1103/PhysRevB.13.5188.

[ref34] Kumagai Y.; Oba F. Electrostatics-Based Finite-Size Corrections for First-Principles Point Defect Calculations. Phys. Rev. B 2014, 89, 195205 10.1103/PhysRevB.89.195205.

[ref35] Freysoldt C.; Neugebauer J.; Van de Walle C. G. Fully Ab Initio Finite-Size Corrections for Charged-Defect Supercell Calculations. Phys. Rev. Lett 2009, 102, 016402 10.1103/PhysRevLett.102.016402. 19257218

[ref36] Murphy S. T.; Hine N. D. M. Anisotropic Charge Screening and Supercell Size Convergence of Defect Formation Energies. Phys. Rev. B 2013, 87, 094111 10.1103/PhysRevB.87.094111.

[ref37] Henkelman G.; Uberuaga B. P.; Jónsson A. A climbing image nudged elastic band method for finding saddle points and minimum energy paths. J. Chem. Phys 2000, 113, 9901 10.1063/1.1329672.

[ref38] Henkelman G.; Jónsson H. Improved tangent estimate in the nudged elastic band method for finding minimum energy paths and saddle points. J. Chem. Phys 2000, 113, 9978 10.1063/1.1323224.

[ref39] Murphy S. T. Tritium solubility in Li_2_TiO_3_ from first principles simulations. J. Phys. Chem. C 2014, 118, 29525–29532. 10.1021/jp508875y.

[ref40] Neilson W. D.; Murphy S. T. DefAP: A Python code for the analysis of point defects in crystalline solids. Comput. Mater. Sci 2022, 210, 111434 10.1016/j.commatsci.2022.111434.

