# Supplementary material for: Tritium Accommodation and Diffusion in Li8PbO6 from First-Principles Simulations
Source: J Phys Chem C Nanomater Interfaces. 2025 Jan 14;129(4):2274–87. doi: 10.1021/acs.jpcc.4c08016 (PMC11789132; doi:10.1021/acs.jpcc.4c08016)
Supplement: Supplementary file 1 — jp4c08016_si_001.pdf [file jp4c08016_si_001.pdf]

Supporting Information for Tritium Accommodation and Diffusion in Li_8PbO_6 from First-principle Simulations

Andrew W. Davies and Samuel T. Murphy*

Department of Engineering, Lancaster University, Bailrigg, Lancaster, LA1 4YW, UK

E-mail: samuel.murphy@lancaster.ac.uk

Tritium Interstitial Reaction Pathways

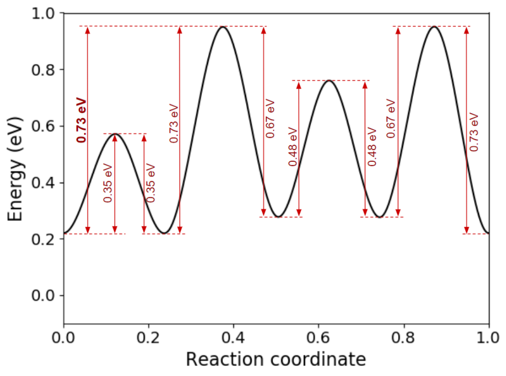

Figure S1: Potential energy surface diagram illustrating the barrier heights for the $c \to c \to b \to b \to c$ diffusion process.

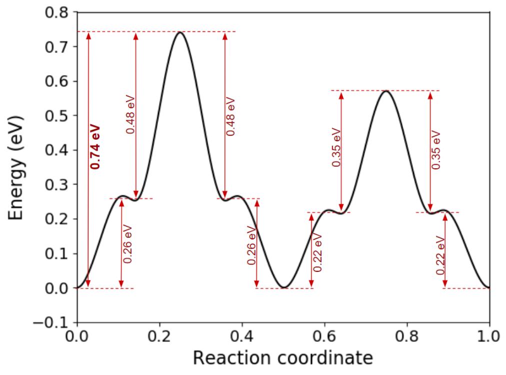

Figure S2: Potential energy surface diagram illustrating the barrier heights for the $a \to b \to b \to a \to c \to c \to a$ diffusion process.

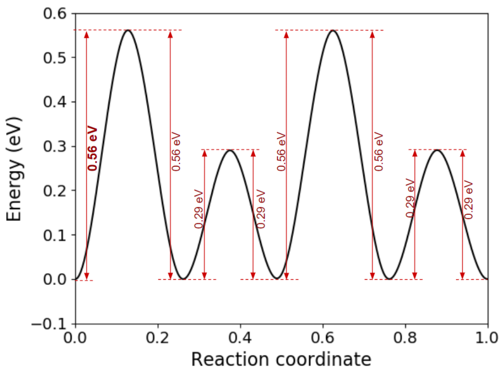

Figure S3: Potential energy surface diagram illustrating the barrier heights for the $a \to a \to \bar{a} \to \bar{a} \to a$ diffusion process.

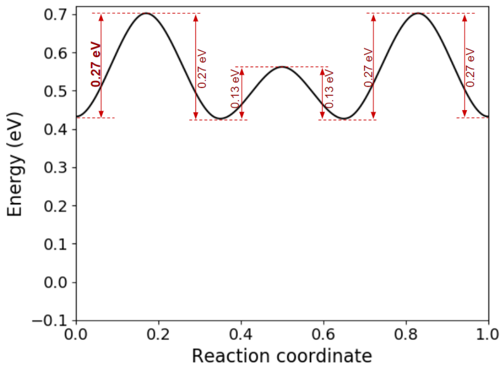

Figure S4: Potential energy surface diagram illustrating the barrier heights for the $d \to \bar{d} \to \bar{d} \to d$ diffusion process.

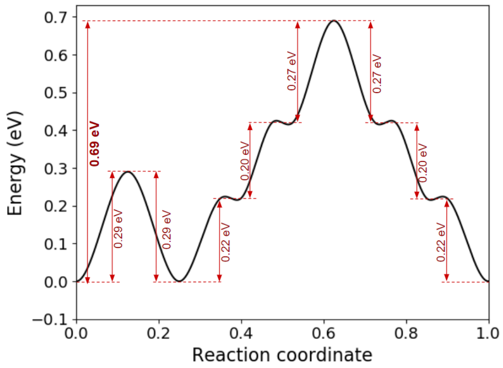

Figure S5: Potential energy surface diagram illustrating the barrier heights for the $\bar{a} \rightarrow a \rightarrow c \rightarrow \bar{d} \rightarrow d \rightarrow \bar{c} \rightarrow \bar{a}$ diffusion process.

Reaction Pathways for Escape from $\{T_i^{+1}:V_{Li}^{-1}\}$ Trapping Sites

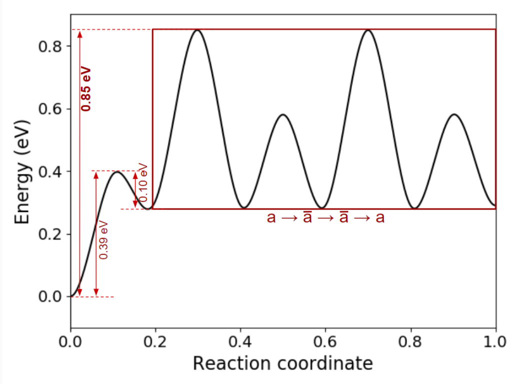

Figure S6: Potential energy surface diagram illustrating the barrier heights for tritium escape from the c' trapping site.

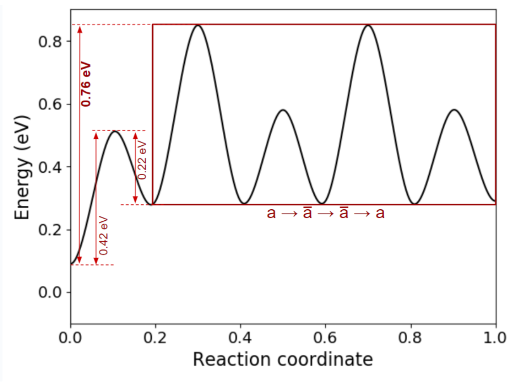

Figure S7: Potential energy surface diagram illustrating the barrier heights for tritium escape from the f' trapping site.

Reaction Pathways for Migration of $\{T_i^{+1}:V_{Li}^{-1}\}$ Defect Cluster

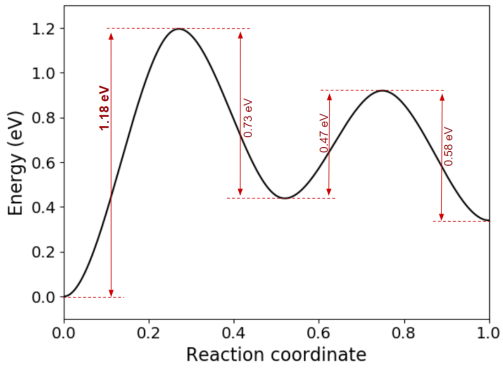

Figure S8: Potential energy surface diagram illustrating the barrier heights along the $T(a')_{Li1} \rightarrow T(f')_{Li2} \rightarrow T(e')_{Li2}$ pathway.

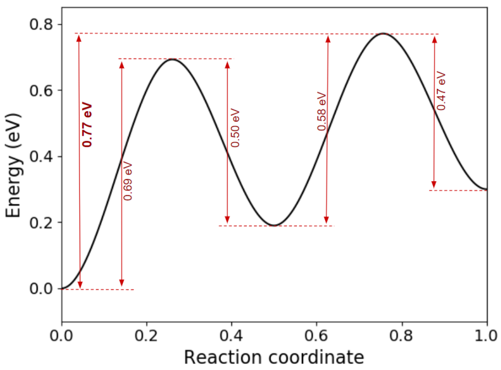

Figure S9: Potential energy surface diagram illustrating the barrier heights along the $T(c')_{Li1} \rightarrow T(e')_{Li2} \rightarrow T(f')_{Li2}$ pathway.

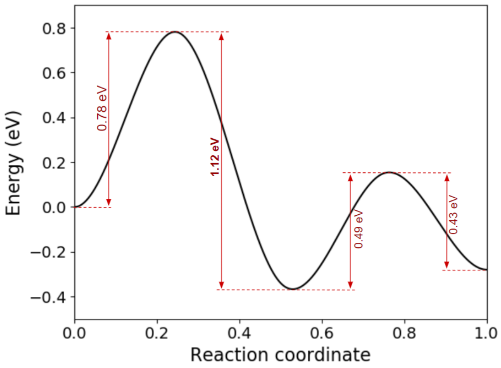

Figure S10: Potential energy surface diagram illustrating the barrier heights along the $T(e')_{Li2} \rightarrow T(a')_{Li1} \rightarrow T(b')_{Li1}$ pathway.

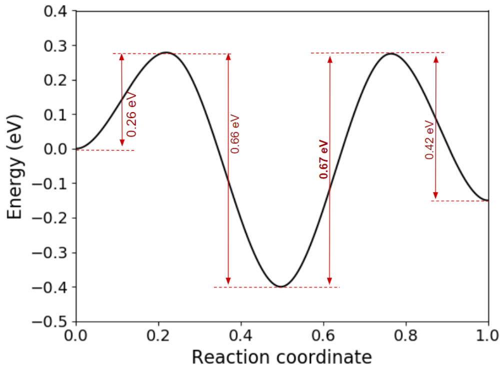

Figure S11: Potential energy surface diagram illustrating the barrier heights along the $\mathrm{T}(f')_{Li2} \rightarrow \mathrm{T}(b')_{Li1} \rightarrow T(\mathrm{d'})_{Li1}$ pathway.
